# Hyperactivity and Differential Gene Expression in *lbx1a^(^^−/^^−)^* Zebrafish Larvae

**DOI:** 10.3390/cells14241980

**Published:** 2025-12-12

**Authors:** Carsten Drepper, Laura Kettenstock, Simon Stöckl, Anna Elsenbach, Carina Lechermeier, Wonhyeok Lee, Susanne Kneitz, Klaus-Peter Lesch, Marcel Romanos, Christina Lillesaar

**Affiliations:** 1 Child and Adolescent Psychiatry, Center of Mental Health, University Hospital Würzburg, 97080 Würzburg, Germanylesch_k@ukw.de (K.-P.L.); romanos_m@ukw.de (M.R.); lillesaar_c@ukw.de (C.L.); 2 Biochemistry and Cell Biology, Biocenter, University of Würzburg, 97074 Würzburg, Germany; susanne.kneitz@uni-wuerzburg.de; 3 Division of Molecular Psychiatry, Center of Mental Health, University Hospital Würzburg, 97080 Würzburg, Germany; 4 Department of Psychiatry and Neuropsychology, School for Mental Health and Neuroscience (MHeNs), Maastricht University, 6229 ER Maastricht, The Netherlands

**Keywords:** brain development, psychiatric disorders, anxiety, ADHD, GABAergic neurons, excitatory/inhibitory imbalance

## Abstract

Lbx1 plays important roles in different processes, including the development of sensory pathways, neuronal cell fate regulation, and muscle cell precursor migration. Genetic variation in the *LBX1* locus has been associated with several human disease conditions, such as idiopathic scoliosis, congenital limb malformation, and neuropsychiatric illness, including attention-deficit/hyperactivity disorder (ADHD) and anxiety disorders. Zebrafish (*Danio rerio*) were used to investigate the behavioral consequences of the loss of function of the two orthologs to the human *LBX1* gene, zebrafish *lbx1a* and *lbx1b*. We observed a consistent locomotor hyperactivity phenotype induced by a novel environment in *lbx1a* mutants. Repeated dark stimuli provoked similar responses in both mutant lines, including the novelty-induced hyperactivity. We performed RNAseq on total RNA isolated from the head region of mutant and wildtype larvae. Several differentially expressed genes were identified, giving more insights into Lbx1 target genes and pathways, which could be relevant regarding the evaluation of zebrafish *lbx1a* or *lbx1b* as a human disease model. Furthermore, the analysis was complemented with a comparison to the expression profile of human LBX1 overexpression in cell culture, revealing a convergence on just two commonly regulated genes, namely alpha-Internexin (*INA*) and Fibrillin-3 (*FBN3*). In conclusion, our findings might further elucidate the multitude of functions of Lbx1 and its involvement in various human disease conditions.

## 1. Introduction

The ladybird homeobox proteins (Lbx) are members of the homeobox transcription factor family containing an engrailed domain and were first described in Drosophila [1]. This family consists of only two members in vertebrates, Lbx1 and Lbx2 [2]. Lbx1 has been shown to play a crucial role in the development of sensory pathways in the spinal cord that relay pain and touch [3] and is required, among other factors, to specify GABAergic cell fate by impacting, e.g., neuropeptide Y (NPY) expression via PAX2 [4]. Moreover, the loss of Lbx1 in mice led to a shift of the cell fate from GABAergic to glutamatergic interneurons in the spinal cord [5]. Similar functions were observed in cell culture [6]. In addition, Lbx1 is important for regulating muscle precursor cell migration and maintaining its migratory potential [7]. In Lbx1 knockout mice specifically, the limb muscles were lacking, other skeletal muscles were developing normally [8]. Later, it was shown that the lateral migration of myoblasts into the distal limb bud were controlled by the phosphorylation of Lbx1, likely through Fgf8 and Erk signaling [9].

Genetic variation at the *LBX1* locus has been associated with several human disease conditions. Idiopathic scoliosis (IS) is the most common spinal deformity in adolescents and is associated with several cosmetic and psychological issues and back pain [10]. Although there are environmental risk factors, a strong genetic contribution has been shown in several genome-wide association studies (GWASs) as well as candidate gene studies [11]. Among several candidate genes, *LBX1* has consistently been identified to be involved in IS by multiple genetic studies and meta-analyses [11,12,13,14,15,16]. One of the strongest associations was found for the SNP rs11190870, which was characterized further in vitro and in zebrafish (*Danio rerio*). The overexpression of human *LBX1* or its zebrafish orthologs *lbx1a* and *lbx1b* caused body axis phenotypes with spine curvature defects in developing zebrafish larvae [17]. A recent study investigated the methylation level in the *LBX1* promoter region in paravertebral muscles in IS patients and controls and found one CpG site locally associated with strong curvature phenotypes [18]. *LBX1* might be involved in the development of IS due to its function in muscle progenitor cell migration and neuronal determination processes [15].

The congenital limb malformation Split-Hand/Foot Malformation type 3 (SHFM3) is associated with tandem duplications in the human *LBX1* locus region on 10q24 [19]. More recently, it was shown that this genomic rearrangement leads to ectopic expression of *Lbx1* and *Btrc* in limb buds in a corresponding mouse model, giving mechanistic insights into the involvement of *LBX1* in SHFM3 [20]. A frameshift mutation in *LBX1* leads to a rare congenital central hypoventilation syndrome in humans due to a disturbed interaction with the transcription factor PHOX2B, which affects a small group of cells in the ventral hindbrain that are central for the hypercapnic reflex, which regulates breathing in response to increased CO_2_ levels [21]. This function of LBX1 is in line with the respiratory distress of *Lbx1* null mice and the Lbx1-dependent development of central respiratory rhythmogenesis in mice [22].

In a large meta-analysis of several GWAS using samples from patients with various anxiety disorders of European origin, a SNP in the *LBX1* region was found to be associated with anxiety disorders using a factor score model [23]. This finding was later replicated in a different population of military service members with an even bigger meta-analysis for *LBX1*, including the previous sample [24]. However, in more recent GWAS meta-analyses, this locus was not replicated [25,26,27]. Finally, whole-exome sequencing (WES) followed by targeted genotyping revealed that the minor A allele of the synonymous SNP rs941909 (G/A) located in exon 1 of *LBX1* perfectly segregated with attention-deficit/hyperactivity disorder (ADHD) in an extended, high-density pedigree previously reported [28]. How *LBX1* might be involved in the development of mental disorders is currently unknown.

Here, we used zebrafish to investigate the behavioral consequences of the loss of function of the two orthologs to the human *LBX1* gene, zebrafish *lbx1a* and *lbx1b*. We used CRISPR/Cas9 to generate null mutants for both genes. Homozygous mutant larvae are viable and were used to characterize behavioral consequences of the loss of *lbx1a* and *lbx1b*, respectively. We detected a robust novel environment-induced hyperactivity phenotype in *lbx1a* mutants. Repeated dark stimuli induced similar responses in both mutant lines, including the novelty-induced hyperactivity. To gain insights into differentially expressed genes upon *lbx1a* knockout, we performed RNAseq on total RNA isolated from the head region of mutant and wildtype larvae. Several differentially expressed genes were identified, giving more insights into Lbx1 target genes and pathways in zebrafish, which could be relevant for further investigations regarding the evaluation of *lbx1a* or *lbx1b* as a human disease model. Furthermore, the analysis was complemented with a comparison to the expression profile of human LBX1 overexpression in cell culture. The combined analysis revealed a convergence on just two commonly regulated genes. This study might help to elucidate the multitude of functions of Lbx1 and its involvement in different disease conditions.

## 2. Materials and Methods

### 2.1. Animal Handling

The zebrafish AB/AB wildtype strain background was used for in situ hybridization (ISH) and generation of the mutant lines. Larvae were raised under standard conditions in Danieau’s solution with or without methylene blue [29] at around 28 °C with a light/dark cycle of 14 h and 10 h, respectively. In case of whole-mount RNA ISH, pigmentation was suppressed using Danieau’s solution containing 0.2 mM 1-phenyl-2-thiourea. Embryos were staged according to [30], manually dechorionated, and fixed in 4% paraformaldehyde (PFA) in 1× phosphate-buffered saline (PBS). Housing and animal handling was performed in accordance with the 3R principles and the local regulations for animal welfare of the University of Würzburg and the District Government of Lower Franconia, Germany.

### 2.2. Cell Culture

The human *LBX1* open-reading frame was PCR-amplified from human first strand cDNA with primers containing restriction enzyme sequences at their 5′ end (Supplementary Table S1) and cloned in a pTarget expression vector containing an HA-tag and CMV promoter (Promega, Madison, WI, USA). HEK293T cells were cultured in T175 flasks with Dulbecco’s Modified Eagle’s Medium (DMEM) with the addition of 1% penicillin/streptomycin solution to prevent bacterial growth as well as 10% fetal calf serum (FCS) and 1% GlutaMAX nutritive supplements at 37 °C with 5% CO_2_. Transfections into HEK293T cells were performed using Lipofectamine 2000 according to the manufacturer’s recommendations (Invitrogen, Carlsbad, CA, USA) at a density of 1 × 10^6^ cells. All transfections were performed in triplicate and processed for total RNA isolation (Qiagen, Hilden, Germany) for RNA sequencing.

### 2.3. Generation of lbx1a and lbx1b Mutant Lines

CRISPR/Cas9 target sites were selected using CHOPCHOP [31]. For synthesis of the single guide RNA (sgRNA), oligonucleotides (Supplementary Table S1) were annealed and ligated with the linearized (*Eco31*I/*Bsa*I, Thermo Fisher Scientific, Waltham, MA, USA) pDR274 vector (Addgene #42250). Integrity of the resulting vectors were verified with Sanger sequencing (Eurofins Genomics, Ebersberg, Germany). Plasmids containing the sgRNA sequence were linearized (*Dra*I, Thermo Fisher Scientific) and in vitro transcribed with a custom-made T7 RNA polymerase (a kind gift from U. Fischer and T. Ziegenhals, University of Würzburg). Purified sgRNA at 100 ng/µL was injected with the Cas9-NLS protein at 300 ng/µL (from *S. pyogenes*, New England Biolabs, Ipswich, MA, USA) into one-cell stage fertilized zebrafish eggs. Sanger sequencing (Eurofins Genomics) was used to confirm successful targeting events (all primer sequences in Supplementary Table S1). Further breeding of the alleles were conducted by outcrossing with AB/AB wildtypes. In order to verify the successful establishment of the mutant lines, gDNA of *lbx1a^(−/−)^* and *lbx1b^(−/−)^* F_3_ mutants was used to PCR amplify the target sites and subsequent sequencing (Eurofins Genomics). For all experiments described below, F_3_ embryos and larvae were used.

### 2.4. Quantitative Real-Time PCR (qPCR)

In this study, 5 dpf-old siblings of *lbx1a* and *lbx1b* mutant lines were used to quantify transcription with quantitative real-time PCR (qPCR). Individual larvae were cut into two parts using a scalpel along a line from below the jaw and above the heart diagonally to the transition from the hindbrain to spinal cord. The tail region, including the spinal cord, was used to extract genomic DNA for genotyping, and the head region, including the central nervous system, the eyes, and the jaw region, was used for total RNA extraction (Qiagen) and subsequent cDNA synthesis (Superscript IV, Invitrogen). Total RNA was isolated on pools of 10 larvae and treated as one biological replicate. Each target gene was analyzed in three technical replicates of each biological sample (*n* = 3) for each genotype. For each run, negative controls were included. Quantification of gene expression changes were performed with the Ct (2^−ΔΔCt^) method using *actb1* and *gapdh* as housekeeping genes (primer sequences in Supplementary Table S1). Group differences were determined by using a one-way ANOVA with the significance level set to 0.05.

### 2.5. Behavior Tests

All behavioral assessments were conducted on larvae at 5 dpf, which were obtained from intercrosses between heterozygous siblings. For each behavioral assay, data collection encompassed 3 to 5 independent experimental replicates. Genotyping procedures were performed as previously described. The genotypes were revealed only after completion of the behavioral tests, thereby ensuring the random distribution of subjects with different genotypes within test arenas and maintaining the experimenter blinded. For both locomotor and activity tracking, the ZebraBox apparatus with the ZebraLab software suite (Version 5.15.0.20, ViewPoint, Lyon, France), operating either in tracking or quantization mode, was utilized. The ZebraBox experimental set-up consisted of an illumination plate situated beneath the test arenas, equipped with an infrared light source and an adjustable white light source as well as an integrated infrared-sensitive camera capturing data at 30 frames per second (fps). Comprehensive descriptions of specific assay arenas and lighting conditions are provided below. Larvae exhibiting gross morphological abnormalities were excluded from behavioral analyses, and data from individual wells showing tracking errors because of technical malfunction were omitted from further data analysis.

#### 2.5.1. Thigmotaxis Assay

Larvae were placed individually in 12-well plates with 1 mL of Danieau’s solution. Swimming tracks were recorded in the dark with infrared illumination and the detection threshold set to 10. Larvae were tracked for a total duration of 40 min, and data analysis was performed in 10 min time bins. To analyze thigmotaxis behavior, each region of interest/well (ROI) was virtually divided into two concentric circular zones: an inner zone with a radius of 7.35 mm and an outer zone with a width of 4 mm. Thigmotaxis behavior was quantified as time spent in the outer zone of the ROI. Locomotor activity was assessed based on total distance swum.

#### 2.5.2. Dark Stimulus-Induced Response Paradigm

The dark stimulus-induced response paradigm addresses the behavioral activity in response to sudden, repetitive changes of light in the shape of dark stimuli after a phase of constant illumination. Larvae were placed individually in 96-well plates with squared wells topped up with Danieau’s solution. The activity (sum of changes in pixel intensity per time unit) of the animals was recorded in the quantization mode of the ZebraLab software with the following defined activity thresholds: freezing, 4; burst, 25. The sensitivity was set to 10, and the data were collected in one s time bins. The test period consisted of a 10 min habituation phase with constant white light (200 lux) followed by a stimuli phase with 10 consecutive 1 s dark stimuli, each separated by a 30 s interval of white light. For the data analyses, the following parameters were calculated: Baseline activity was calculated by the average activity of the last 10 s of the habituation phase. Total peak activity was obtained by the sum of the activity values of the three seconds directly after application of the dark stimulus. Cumulative total peak activity was calculated by the cumulative sum of the 10 total peak activity values. Activity at the first 10 s of the experiment was calculated as the sum of the activity of the first 10 time bins. Similarly, activity during habituation was assessed after the initial 10 s and before the baseline activity (11 s to 589 s).

### 2.6. RNA Sequencing Analysis

Total RNA was isolated from pools of ten 5 dpf animals of *lbx1a* ^(−/−)^ and ^(+/+)^ siblings as described above. A 2100 Bioanalyzer and the RNA 6000 Nano kit (Agilent Technologies, Santa Clara, CA, USA) were used to check RNA quality. The RNA integrity number (RIN) for all samples was above 9.3. An amount of 500 ng of total RNA was used to prepare sequencing-ready DNA libraries using oligo-dT capture beads and the TruSeq Stranded mRNA Library Preparation Kit (^1/2^ volume, Illumina, San Diego, CA, USA). After completing 14 cycles of PCR amplification, the average size of the barcoded DNA libraries was determined to be in the range of ~310 bp on Agilent DNA 1000 Bioanalyzer microfluidic chips (Agilent Technologies, Santa Clara, CA, USA). Sequencing of pooled libraries was performed at ~22 million reads per sample in single-end mode with a read length of 100 nt with a P2 sequencing kit on the NextSeq 2000 platform (Illumina). Demultiplexed FASTQ files were generated with bcl2fastq2 (Version 2.20.0.422, Illumina). RNAseq data were further processed using the DESeq2 (Version 1.40.2) [32] as well as a gene ontology and pathway enrichment analysis using clusterProfiler [33] and pathview [34] (https://www.bioinformatics.com.cn/srplot, accessed on 21 May 2025). Data were visualized using SRplot [35] (https://www.bioinformatics.com.cn/srplot, accessed on 21 May 2025).

### 2.7. Data Analysis and Statistics

Behavioral data generated from the Zebralab software (Viewpoint) were processed with Excel (Microsoft). Behavior data analysis and visualization were performed with the statistic software JASP (version 0.95.4) [36]. The Shapiro–Wilk test was used to test for normal distribution. Depending on distribution, an independent samples *t*-test or Mann–Whitney test was used to calculate group differences. The level of significance was set to 0.05. Appropriate sample sizes were determined using the software G*Power 3.1.9.4 [37,38].

## 3. Results

### 3.1. Both Orthologs of the Human LBX1 Gene Are Showing Distinct Expression Patterns in Zebrafish Larvae

There are two orthologs of the human *LBX1* gene (ENSG00000138136) present in zebrafish [2,39,40]. The human gene is located on chromosome 10 and consists of 2 exons, coding for a protein of 281 aa. The two zebrafish orthologs are located on chromosome 13 (*lbx1a*, ENSDARG00000018321, encoding a protein of 269 aa) and chromosome 1 (*lbx1b*, ENSDARG00000018611, encoding a protein of 265 aa), respectively. The zebrafish sequences are matching the human protein sequence to 76.21% for Lbx1a and 61.51% for Lbx1b. The expression patterns of *lbx1a* and *lbx1b* were published earlier using ISH [39]. We were able to confirm expression of both genes at several developmental stages (24, 36, and 48 hpf) and noted common and distinct expression domains (Supplementary Figure S1A,B). Although both genes are expressed in the spinal cord, the developing hindbrain and the pectoral fin muscles, there are subtle differences. For instance, migratory muscle precursors are labeled for *lbx1a* expression at 24 hpf. This group of cells is not labeled for *lbx1b* expression (Supplementary Figure S1A,B). The expression pattern in the hindbrain shows subtle differences. Both genes are expressed in the dorsal hindbrain; however, *lbx1b* is present slightly more dorsal than *lbx1a* at 24 and 36 hpf. Further, from 36 hpf and onwards, *lbx1b* expression is additionally visible in the presumable upper rhombic lip. For *lbx1a*, a distinct labeling is visible laterally in a structure caudal to the eye, presumably the trigeminal ganglion. Together, these findings suggest that the two paralogs are detected in distinct or only partially overlapping cell populations. The expression differences suggest that a subfunctionalization between the two paralogs may have occurred during evolution. This assumption is strongly supported by the findings in previous studies [40].

### 3.2. Generation of lbx1a and lbx1b Loss-of-Function Lines

**Figure 1:**
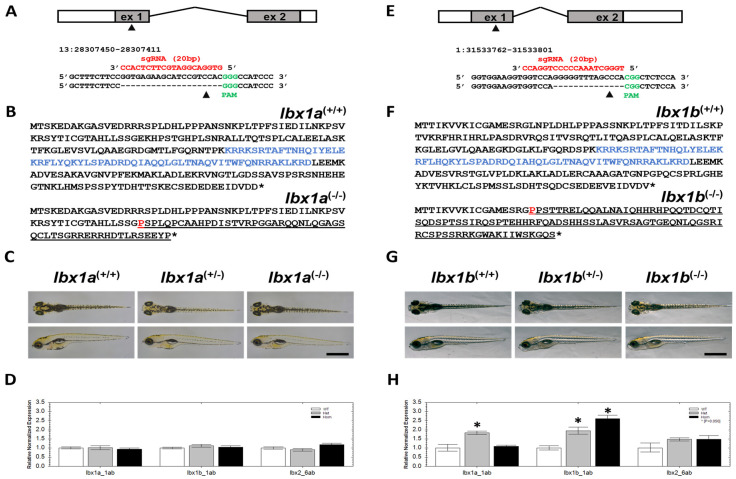
The generation of *lbx1a* and *lbx1b* loss-of-function lines. (**A**) The targeting site in exon 1 of the *lbx1a* gene and the sequence of the guide RNA used. (**B**) The wildtype protein sequence of Lbx1a (upper sequence) and truncated protein sequence after the targeting event (lower sequence). (**C**) Phenotypic appearances of *lbx1a*^(+/+)^, *lbx1a*^(+/−)^, and *lbx1a*^(−/−)^ animals. Upper row dorsal view, lower row side view. Note that no obvious phenotype is visible. (**D**) The relative normalized expression of *lbx1a*, *lbx1b*, and *lbx2* in *lbx1a*^(+/+)^, *lbx1a*^(+/−)^, and *lbx1a*^(−/−)^ animals using qPCR. (**E**) The targeting site in exon 1 of the *lbx1b* gene and the sequence of the guide RNA used. (**F**) The wildtype protein sequence of Lbx1b (upper sequence) and truncated protein sequence after the targeting event (lower sequence). (**G**) Phenotypic appearances of *lbx1b*^(+/+)^, *lbx1b*^(+/−)^, and *lbx1b*^(−/−)^ animals. Upper row dorsal view, lower row side view. Note that no obvious phenotype is visible. (**H**) The relative normalized expression of *lbx1a*, *lbx1b*, and *lbx2* in *lbx1b*^(+/+)^, *lbx1b*^(+/−)^, and *lbx1b*^(−/−)^ animals using qPCR. (**B**,**F**) The sequence highlighted in blue indicates the homeobox domain. Group differences were calculated with ANOVA test, *p* > 0.05 *.

Due to differences in the expression pattern and previously characterized functional roles in zebrafish, we decided to generate stable null mutants for both paralogs in zebrafish in order to characterize functional roles for both *lbx1* genes separately. Several mutant lines were generated using CRISPR/Cas9, and one line for each gene was screened for morphological and behavioral phenotypes. The *lbx1a* line contains a 20 bp deletion in exon 1 (Figure 1A and Figure S2A). For *lbx1b*, we selected a line with a 14 bp deletion in exon 1 (Figure 1E and Figure S2B). The deletion mutations are predicted to result in a frameshift and introduction of a premature stop codon in both cases, which eventually leads to a termination of translation upstream of the homeobox domain, potentially generating protein fragments of 115 and 113 amino acids, respectively (Figure 1B,F). As only small N-terminal parts of the proteins remain, which are entirely lacking in the important domains for transcription factor activity, we think it is highly unlikely that any residual Lbx1 function is emanating from the two mutated alleles. Therefore, we assume that both lines are potential null alleles.

We found no gross morphology differences in *lbx1a* (Figure 1C) and *lbx1b* (Figure 1G) mutants. No significant differences in yolk diameter, body length, and head size of mutant compared to wildtype siblings were detected. Likewise, acridine orange staining in mutants and wildtype larvae revealed no differences in cell apoptosis (Supplementary Figure S3A,B). In addition, there was no indication for early lethality in both mutant lines (Supplementary Figure S4A,B). Next, we quantified the expression of *lbx1a*, *lbx1b*, and *lbx2* transcripts in both mutant lines (Figure 1D,H). Whereas in *lbx1a* mutants no expression differences in *lbx1a*, *lbx1b*, and *lbx2* transcript levels were detected (Figure 1D), a significant increase in *lbx1a* expression was found in *lbx1b*^(+/−)^ animals (Figure 1H). In *lbx1b*^(−/−)^ animals, the expression level of *lbx1a* was indistinguishable from *lbx1b*^(+/+)^ animals. The *lbx1b* transcript levels were significantly increased in both *lbx1b*^(+/−)^ and *lbx1b*^(−/−)^ animals, indicating a potential compensation mechanism due to the loss of functional Lbx1b protein (Figure 1H). The expression of *lbx2* was unchanged in *lbx1b*^(−/−)^ mutant animals (Figure 1H). Taken together, the wildtype-like appearance of *lbx1a* and *lbx1b* mutant lines suggests that each of the *lbx1* genes are not critical for anatomical development and cell survival.

### 3.3. lbx1a and lbx1b Mutant Lines Show Differential Motility Phenotypes

**Figure 2:**
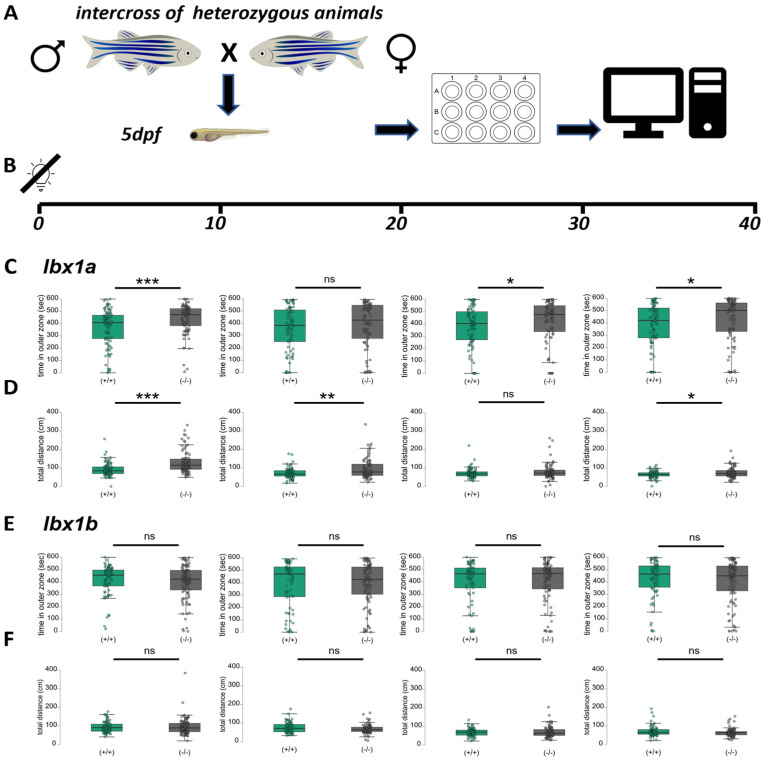
Thigmotaxis and hyperactivity in *lbx1a*^(−/−)^ but not in *lbx1b*^(−/−)^ mutants. (**A**) The experimental outline and test arena for thigmotaxis place preference with the virtual division of analysis zones in the 12-well format. The central zone indicates exposed area and the outer zone defensive preferences. (**B**) An experimental outline for the 40 min assay and analysis in groups of 10 min. (**C**) Thigmotaxis behavior in the *lbx1a* line shown as time in outer zone. Note that in the first 10 min significant differences in thigmotaxis behavior between genotypes are visible (Mann–Whitney *p* < 0.001, effect size 0.312 ± 0.086), which later decreases. (**D**) Hyperactivity in the *lbx1a* line shown as total distance traveled in 10 min. Note that in the first 10 min, strong differences between genotypes are visible (Mann–Whitney *p* < 0.001, effect size 0.455 ± 0.086), which later decreases. (**E**) The place preference analysis in the *lbx1b* line shown as time in outer zone. Note that no significant differences between genotypes are visible. (**F**) No hyperactivity in *lbx1b*^(−/−)^ animals detectable. Data from 4 independent experiments with in total 80 to 100 animals per genotype. Data analyzed with a two-tailed Mann–Whittney test with *p* < 0.05 *, *p* < 0.01 ** and *p* < 0.001 ***.

To investigate the behavioral phenotypes of the *lbx1a* and *lbx1b* mutant lines, we performed locomotor tracking in 5 dpf larvae. Therefore, we placed larvae individually in a 12-well plate and recorded locomotor activity for 40 min in the dark directly without habituation time (Figure 2A,B). To detect a potential thigmotaxis phenotype, we defined two concentric circular zones (Figure 2A) and quantified the wall-hugging behavior, which is defined as time spent close to the wall of the well. This behavior is generally assumed to reflect anxiety-like behavior [41]. Locomotor activity levels were analyzed in 10 min time bins for total distance (in cm) and time spent in outer zone for ^(+/+)^ and ^(−/−)^ animals (Figure 2C–F). For *lbx1a*, significant differences in both parameters were detected in the first 10 min, as indicated in the left charts in *lbx1a*^(−/−)^ animals (Figure 2C,D). From the second time bin (10–20 min) onwards, the behavioral differences between *lbx1a*^(+/+)^ and *lbx1a*^(−/−)^ animals were becoming weaker although still significant (Figure 2C,D). In contrast, in *lbx1b* mutant animals, no behavioral differences were detected (Figure 2E,F). The thigmotaxis behavior was not changed for *lbx1b*^(−/−)^ mutants compared to wildtype siblings (Figure 2E). Additionally, the total distance traveled showed no differences (Figure 2F). This characterization of *lbx1a* and *lbx1b* mutant behavior in larvae points towards a strong novel tank phenotype in *lbx1a*^(−/−)^ animals, which results in hyperactivity early during the first 20 min in this experiment, which later returns to normal levels and a lack of phenotype in *lbx1b*^(−/−)^ animals in this assay. This clearly demonstrates distinct functions in the early development of zebrafish larvae for each paralog, which translates into a distinct behavioral phenotype in *lbx1a*^(−/−)^ animals.

### 3.4. lbx1a and lbx1b Mutants React Similarly to Repeated Dark Stimuli

In the next behavioral assay, we challenged the animals of both lines by applying repeated dark stimuli. As the *lbx1a*^(−/−)^ larvae display a strong novel tank phenotype in the dark, we hypothesized that a set of repeated visual dark stimuli could evoke a stronger phenotype as seen for the novel tank situation in the previous experiment compared to wildtype animals.

**Figure 3:**
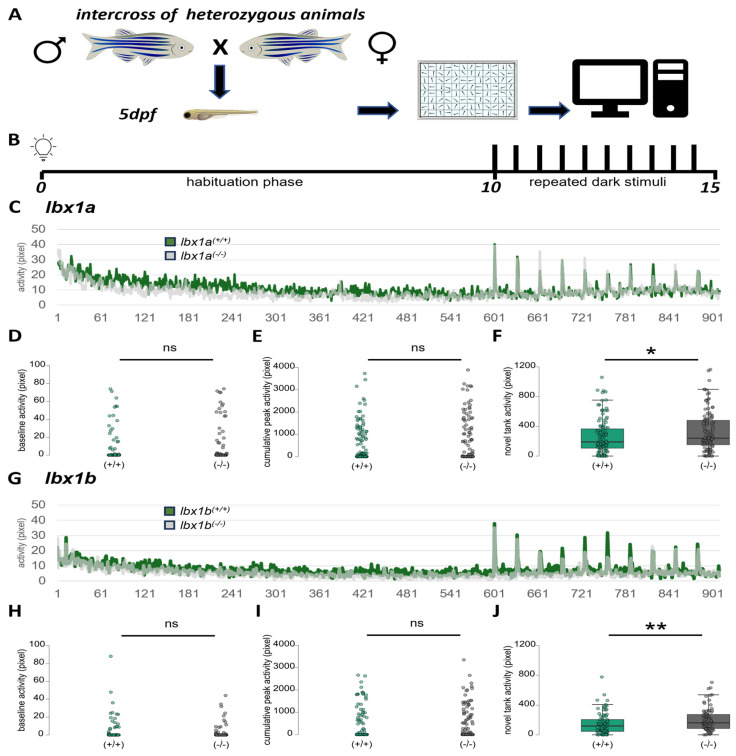
Similar reaction in repeated dark stimulus assay in *lbx1a*^(−/−)^ and *lbx1b*^(−/−)^ mutants. (**A**) The experimental outline and test arena for the repeated dark stimulus assay in a 96-well format. (**B**) The experimental outline for the 15 min assay with a 10 min habituation phase and 10 stimuli for one second and 30 s of interstimulus interval. (**C**) The activity plot over the experiment. Activity is measured in pixel intensity change and is shown for *lbx1a*^(+/+)^ animals (green) and *lbx1a*^(−/−)^ animals (gray). Note the strong reaction to the dark stimuli. (**D**) Baseline activity 10 s prior to the first stimulus. (**E**) The cumulative peak activity of the first 3 s after each peak summed up over all 10 stimuli. (**F**) The activity of the first 10 s of the experiment. Note the increased activity of the *lbx1a*^(−/−)^ animals (Mann–Whitney *p* = 0.037, effect size 0.160 ± 0.076). (**G**) The activity plot over the experiment. Activity is measured in pixel moved and is shown for *lbx1b*^(+/+)^ animals (green) and *lbx1b*^(−/−)^ animals (gray). (**H**) Baseline activity 10 s prior to the first stimulus. (**I**) The cumulative peak activity of the first 3 s after each peak summed up over all 10 stimuli. (**J**) The activity of the first 10 s of the experiment. Note the increased activity of the *lbx1b*^(−/−)^ animals (Mann–Whitney *p* = 0.006, effect size 0.248 ± 0.089). Data analyzed with a two-tailed Mann–Whitney test with *p* < 0.05 * and *p* < 0.01 **.

To test this, we placed individual larvae in single wells of a 96-well plate (Figure 3A) and recorded activity during constant illumination for 10 min (habituation phase) and presented ten dark stimuli of one second interspersed with 30 s of constant illumination (Figure 3B). The activity plots for each genotype revealed a strong reaction towards the dark stimuli, which declines over the course of the experiment due to habituation to repeated stimuli in both mutant lines (Figure 3C,G). Firstly, we determined the baseline activity, which we defined as the average activity of the 10 s prior to the first stimulus. In neither *lbx1a*^(−/−)^ nor *lbx1b*^(−/−)^ animals, we detected differences compared to the wildtype (Figure 3D,H). Next, we analyzed the total peak activity by summing up the activity of the three seconds directly after each dark stimulus and built the cumulative peak activity for each genotype (Figure 3E,I). Contrary to our hypothesis, we did not detect any differences for either of the homozygous mutants compared to wildtype animals (Figure 3E,I). Both lines react similarly to the dark stimuli applied in this experiment. However, by investigating the first 10 s of the habituation phase, we noted an increased activity in both mutant lines *lbx1a*^(−/−)^ and *lbx1b*^(−/−)^ compared to the wildtype littermates (Figure 3F,J), probably due to the sudden sensory input at the beginning of the experiment. The analysis of the remaining habituation phase before the stimuli did not show any differences between mutant and wildtype littermates in both mutant lines (Supplementary Figure S5A,B). Taken together, this experiment in the light shows a similar phenotype in both lines. Each line reacts strongly to the repeated dark stimuli, which declines due to habituation. Similarly, the increased activity at the beginning of the habituation phase was detected in both mutant lines.

### 3.5. Differentially Expressed Transcripts in the Head Region of lbx1a^(−/−)^ Larvae

**Figure 4:**
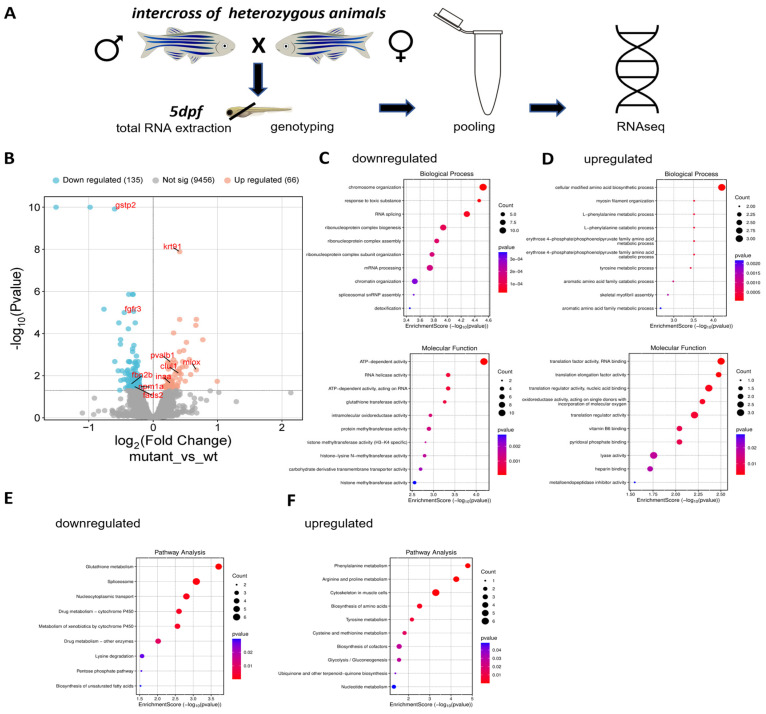
Differentially expressed genes in the head region of *lbx1a*^(−/−)^ larvae. (**A**) The experimental outline of the expression profiling. Note the site of separation of the head and trunk region of the animals. The head region was used for expression profiling and the trunk for genotyping. (**B**) The volcano plot of the experiment. One hundred thirty-five genes were downregulated, sixty-six upregulated. (**C**) The GO term enrichment analysis of the downregulated genes for biological processes (**upper panel**) and molecular function (**lower panel**). (**D**) The GO term enrichment analysis of the upregulated genes for biological processes (**upper panel**) and molecular function (**lower panel**). (**E**) The pathway enrichment analysis for the downregulated genes. (**F**) The pathway enrichment analysis for the upregulated genes.

In order to find differentially expressed genes that could give insights into the underlying biology of the behavioral phenotypes detected in the *lbx1a* line, we decided to conduct an RNAseq experiment in *lbx1a*^(+/+)^ and *lbx1a*^(−/−)^ animals (Figure 4A). The list of differentially expressed genes (DEGs) was ranked according to the adjusted *p*-values. In total, 201 genes were differentially expressed with an adjusted *p*-value below 0.05 in *lbx1a* mutants compared to the control siblings. The log2FC values ranged from ≤−1.51 downregulated DEGs in mutants to ≥+0.99 upregulated DEGs in mutants, which cluster nicely according to genotype (Supplementary Figure S6). The volcano plot shows the relation of up- and downregulated transcripts in this experiment (Figure 4B). First, the expression differences of *lbx1a* and other ladybird homeobox family members were evaluated in more detail in mutants compared to *lbx1a*^(+/+)^ animals. *Lbx1a* displayed only a slightly elevated expression level (+0.59, nom *p*-value 0.00, adj *p*-value na), which might be due to a potential compensatory effect of the loss of function of the Lbx1a protein in the tissues investigated. Expression difference for *lbx1b* was not detected (+0.21, nom *p*-value 0.43, adj *p*-value na). Similarly, *lbx2* (+0.16, nom *p*-value 0.26, adj *p*-value na) did not show expression differences (Supplementary Figure S7). Taken together, no differences in the expression of the other ladybird homeobox genes but a very mildly elevated expression of the target gene itself was detected in *lbx1a^(^*^−/−)^ animals, thus largely confirming our qPCR results before. The gene list was separated into 135 downregulated genes (ranging from −1.51 to −0.20, Supplementary Table S2) and 66 upregulated genes (ranging from 0.99 to 0.17, Supplementary Table S3) and evaluated separately in a GO term enrichment analysis. Among the downregulated genes, a significant enrichment of GO terms was detected for biological processes related to chromosome organization (GO:0051276), response to toxic substance (GO:0009636) and RNA splicing (GO:0008380), and several other RNA-related GO terms driven by the same gene set: *ptbp1b, snrpf*, *ptbp1a*, *snrpd2*, *prpf8*, *prpf19*, *hmga1a*, *snrnp200*, and *cirbpa* (Figure 4C upper panel, Supplementary Table S4). The same trend was seen in the category molecular function, where the top hit was ATP-dependent activity (GO:0140657) and RNA helicase activity (GO:0003724) (Figure 4C lower panel, Supplementary Table S5). The cellular component analysis showed the downregulation of genes involved in the spliceosomal complex (GO:0005681), histone methyltransferase complex (GO:0035097), nuclear envelope (GO:0005635), and nuclear pore (GO:0005643) (Supplementary Figure S8A, Supplementary Table S6). Unexpectedly, no neuronal GO terms were enriched at all in this analysis. In summary, the GO term enrichment analysis showed among the mapped downregulated genes a potential chromosome organization phenotype and an altered RNA processing, splicing, or stability phenotype in *lbx1a*^(−/−)^ animals. Manual screening of the downregulated genes resulted in some interesting candidate genes. Among these were *fgfr3* (fibroblast growth factor receptor 3, −0.45, adj *p*-value 0.00), which is involved in bone formation [42,43]; *fbn2b* (fibrillin 2b, −0.35, adj *p*-value 0.03), which is a candidate gene for Marfan syndrome and scoliosis in humans [11,44,45,46]; *nav1* (neuron navigator 1, −0.39, adj *p*-value 0.03 [47]); *dusp1* (dual specificity phosphatase 1, −0.37, adj *p*-value 0.04); *prdx1* (peroxiredoxin 1, −0.36, adj *p*-value 0.01); and *ranbp1* (RAN binding protein 1, −0.21, adj *p*-value 0.03 [48,49]. Similarly, we performed a GO term enrichment analysis for 66 upregulated genes in *lbx1a*^(−/−)^ animals. An enrichment of GO terms was detected for biological processes related to the cellular modified amino acid biosynthetic process (GO:0042398), which was driven by the creatine kinase genes *ckma*, *ckmb*, *ckmt2b*, and myosin filament organization (GO:0031033) (Figure 4D upper panel, Supplementary Table S7). In the molecular function category translation factor binding, RNA binding was enriched (GO:0008135) as well as several other related GO terms driven by the same gene set: *eef1a1a*, *eef1da*, and *eif3s6ip* (Figure 4D lower panel, Supplementary Table S8). The GO terms for the dynactin complex (GO:0005869), intermediate filaments (GO:0005882), and myofibril (GO:0030016) were significant in the section cellular component (Supplementary Figure S8B, Supplementary Table S9). No significant enrichment for GO terms related to neuronal, glial, synaptic function, or bone formation were detected. However, manual screening of the upregulated genes revealed some interesting candidate genes. The top hit in the list of upregulated genes is the *si:ch1073-464p5.5* gene (+0.99, adj *p*-value 0.00), which is an uncharacterized protein with a thioredoxin domain and no mammalian ortholog; *ttc36* (tetratricopeptide repeat domain 36, +0.52, adj *p*-value 0.00) is a molecular chaperone involved in different processes, including hippocampal neuron function and learning and memory [50]; *snorc* (secondary ossification center associated regulator of chondrocyte maturation, +0.41, adj *p*-value 0.00) is a cartilage-specific transmembrane proteoglycan [51,52]; and *pvalb1* (parvalbumin 1, +0.27, adj p-value 0.00), *inaa* (internexin neuronal intermediate filament protein, alpha a, +0.27, adj p-value 0.02), *pfn1* (profilin 1, +0.24, adj *p*-value 0.00), and *pvalb2* (parvalbumin 2, +0.23, adj *p*-value 0.02) are important neuronal expressed genes. In addition to the GO term enrichment analysis, we performed on the gene sets for down- and upregulated genes a pathway enrichment analysis. Among the downregulated genes, glutathione metabolism (dre00480), spliceosome (dre03040), and nucleocytoplasmic transport (dre03013) were enriched (Figure 4E, Supplementary Table S10). Among the upregulated genes, phenylalanine metabolism (dre00360), arginine and proline metabolism (dre00330), and cytoskeleton in muscle cells (dre04820) were enriched (Figure 4F, Supplementary Table S11). Taken together, no obvious gene expression differences were identified that could directly explain the behavioral differences that we have detected (see above). Given the broad spectrum of disorders where Lbx1 is involved, this gene and the pathway lists could be of relevance for disorders, where the *lbx1a*^(−/−)^ mutant line can be used to investigate other aspects of Lbx1 function.

### 3.6. Overexpression of Human LBX1 in Cell Culture Identifies Differentially Expressed Transcripts

**Figure 5:**
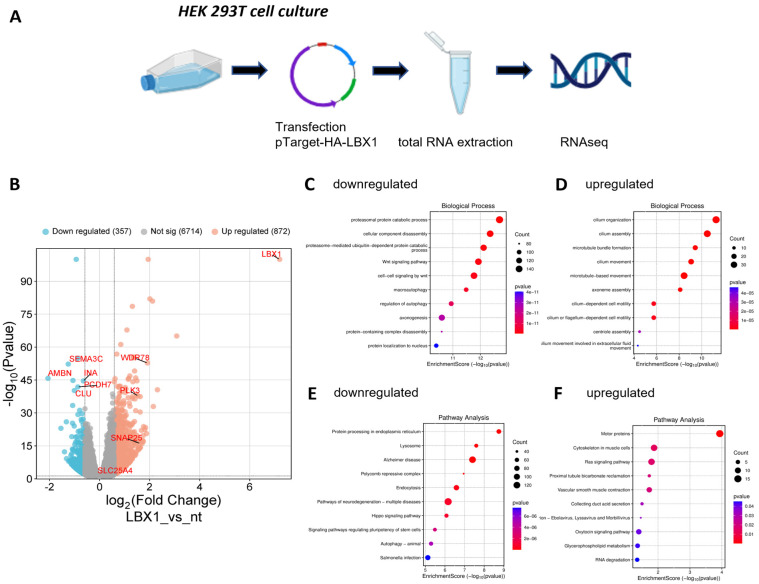
The overexpression of human LBX1 in cell culture and identification of differentially expressed genes. (**A**) The experimental outline. Human LBX1 was overexpressed in HEK293T cells and total RNA extracted and sequenced. (**B**) The volcano plot of the experiment. Three hundred fifty-seven genes were downregulated, eight hundred seventy-two upregulated. (**C**) The GO term enrichment analysis of the downregulated genes for biological processes. (**D**) The GO term enrichment analysis of the upregulated genes for biological processes. (**E**) The pathway enrichment analysis for the downregulated genes. (**F**) The pathway enrichment analysis for the upregulated genes.

To obtain independent information about gene targets of LBX1, we complemented our expression study in zebrafish with an overexpression cell culture experiment. We cloned the human LBX1 open-reading frame with a N-terminal HA-tag into the pTarget expression vector and transfected the human embryonic kidney cell line HEK293T (Figure 5A).

After confirming successful LBX1 expression, we extracted RNA and performed RNAseq similarly to the previous experiment with the zebrafish mutants. The analysis of the data revealed that we strongly overexpressed *LBX1* (log2FC 7.192, *p*-value 0, adj *p*-value 0) compared to the endogenous expression (Supplementary Table S12). We set a threshold of 1.5-fold change (log2FC 0.58 with adj *p*-value 0.05). We set this higher threshold compared to the zebrafish experiment due to the fact that we wanted to restrict the analysis to the very strongly regulated transcripts. With these settings, we identified 872 upregulated and 357 downregulated genes (Figure 5C). Based on the downregulated genes, GO term enrichment analysis identified proteasomal catabolic processes (GO:0010498), the WNT signaling pathway (GO:0016055), and axonogenesis (GO:0007409) as biological processes affected due to the overexpression of human *LBX1* (Figure 5D, Supplementary Table S13). GO term enrichment analysis for the upregulated genes identified an enrichment of genes involved in cilium biology (GO:0044782) and microtubule-based movements (GO:0007018) (Figure 5E, Supplementary Table S14). Next, we looked at pathway enrichment. Among the downregulated genes, we found an enrichment of pathways related to neurodegeneration (hsa05022) and Alzheimer’s (hsa05010), protein processing in the endoplasmic reticulum (hsa04141) and the Hippo signaling pathway (hsa04390) and signaling pathways regulating the pluripotency of stem cells (hsa04550) (Figure 5F, Supplementary Table S15). The pathway analysis for the upregulated genes resulted in motor proteins (hsa04814) and cytoskeleton in muscle cells (hsa04820) as top hits as well as the ras signaling pathway (hsa04014) and oxytocin signaling pathway (hsa04921) (Figure 5F, Supplementary Table S16).

### 3.7. Analysis of Regulated Genes in Zebrafish and Cell Culture Revealed Putative Targets of Lbx1

**Figure 6:**
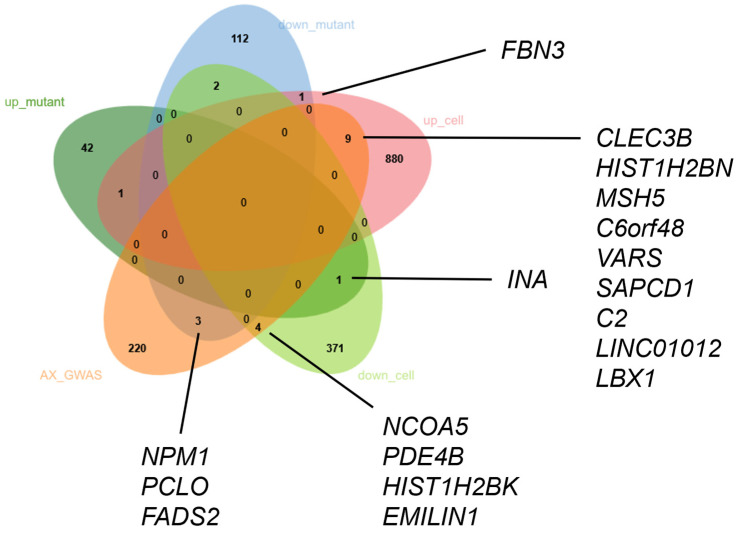
The combined analysis of differentially expressed genes in both experimental set-ups. A Venn diagram showing the amount of overlap of the downregulated genes in *lbx1a^(−/−)^* animals (down_mutant, blue), upregulated in *lbx1a^(−/−)^* animals (up_mutant, dark_green), downregulated in HEK293 with *LBX1* overexpression (down_cell, light green), upregulated in *LBX1* overexpression (up_cell, red), and GWAS candidate genes for anxiety disorders (AX_GWAS, orange). Overlapping genes in interesting categories are highlighted. The complete gene list can be found in Supplementary Table S19.

In order to identify Lbx1 target genes based on both expression experiments, we combined both RNAseq analyses, determined the human orthologs of the regulated zebrafish genes in the *lbx1a*^(−/−)^ mutant (Supplementary Tables S17 and S18) using the g:Orth function in g:Profiler [53], and determined the overlap of the resulting gene lists using jvenn [54]. Assuming that Lbx1 target genes should show a differential direction of regulation if the regulator is absent (zebrafish *lbx*^(−/−)^ model) or overexpressed (*LBX1* overexpression in cell culture), we focused on upregulated in mutants and downregulated in cell culture (Lbx1 as a putative negative regulator) and downregulated in mutants and upregulated in cell culture (Lbx1 as a putative positive regulator), respectively (Figure 6, Supplementary Table S19). Using this strategy, we found only two transcripts fulfilling these criteria. *FBN3* is upregulated upon the overexpression of *LBX1* in cell culture and downregulated in the *lbx1a*^(−/−)^ mutant (Figure 6). Similarly, INA is upregulated in *lbx1a*^(−/−)^ mutants and downregulated in *LBX1* overexpression in cell culture. Next, we explored these results further with comparison to the recently published lists of GWAS candidate genes for anxiety disorders [23,25,26,27] to identify putative genes that could be involved in the anxiety-like phenotype we detected in the *lbx1a*^(−/−)^ mutant line or cell culture experiment. Using jvenn, we found an overlap of the GWAS genes with nine genes upregulated in cell culture (*CLEC3B*, *HIST1H2BN*, *MSH5*, *C6orf48*, *VARS*, *SAPCD1*, *C2*, *LINC01012*, and *LBX1*), with four genes downregulated in cell culture (*NCOA5*, *PDE4B*, *HIST1H2BK*, and *EMILIN1*), and with three genes downregulated in the *lbx1a*^(−/−)^ mutant line (*NPM1*, *PCLO*, and *FADS2*). There was no overlap of the regulated genes, in neither cell culture nor the mutant zebrafish line, with the recently published ADHD Meta-GWAS [55] (Supplementary Figure S10). In summary, although the overlap of differentially expressed transcripts in both experimental systems is limited to only two transcripts, both transcripts could be relevant target genes for Lbx1 function and could give deeper insight into disease mechanisms where Lbx1 could be involved.

## 4. Discussion

To gain knowledge about the potential involvement of LBX1 in human disorders, we generated two mutant lines, which allowed us to investigate the contribution of *lbx1a* and *lbx1b* to potential behavioral consequences. In the dark, *lbx1a* mutant animals displayed a hyperactivity and thigmotaxis phenotype, and the *lbx1b* line was lacking a phenotype. In the second behavioral setting, in the light with dark stimuli, both lines displayed a similar phenotype, a novel tank-elicited hyperactivity at the beginning of the experiment. The reactions to the dark stimuli were in both lines indistinguishable to wildtype littermates. Expression profiling in the *lbx1a* mutant as well as in cell culture delivered a multitude of regulated transcripts, which could be useful in assessing Lbx1 function in several human disorders. These results lay the foundation for a further and deeper exploration of Lbx1 function in zebrafish.

Subtle differences between both genes were revealed with ISH (Supplementary Figure S1). The expression patterns of zebrafish *lbx1a*, *lbx1b*, and *lbx2* have been characterized before [39,56]. The expression of Lbx family members were found in the developing neural tube, including the hindbrain and spinal cord. In limb buds, a similar function as in mice was suggested [56,57]. *Lbx1a* expression was found in migratory muscle precursor cells [39,58]; however, this group of cells are devoid of *lbx1b* expression [39]. In our analysis, we found a similar distribution. The more dorsal expression of *lbx1b* compared to *lbx1a* especially suggests a subfunctionalization of Lbx1 in zebrafish during evolution. A similar distribution was suggested by a previous study [40], where the *lbx1a* expression was found in dl4-6 interneurons, whereas *lbx1b* expression was detected in spinal cord progenitor cells in the dP4 and potentially also the dP5 domain [40]. Therefore, based on the expression pattern of *lbx1a* and *lbx1b*, one should expect different behavioral phenotypes from the knockout of each individual gene. We therefore decided to generate null mutants for both genes and analyzed their behavior. As *lbx1a^(−/−)^* animals display increased locomotion and a thigmotaxis phenotype we assume that this could be due to the proposed role of *lbx1a* in specifying GABAergic neuron cell fate in specific regions of the brain [4] and acting at the switch of excitatory to inhibitory neurons in the spinal cord. Similarly to mouse Lbx1, zebrafish *lbx1a* null mutants show less inhibitory and more excitatory neurons in the spinal cord [5,40]. A reduction of inhibitory neurons was also detected in *lbx1b* null mutants but without the increase in excitatory neurons [40], which could explain the difference between both mutants in showing the hyperactivity and thigmotaxis phenotype (Figure 2C–F). The novel tank hyperactivity of both mutant lines in the second behavioral assay could be explained by a reduced number of inhibitory neurons, which could translate into increased reaction to novel environments (Figure 3F,J). From the similar strong reaction to the dark stimuli in both lines, we can conclude that the differences in excitatory neuron formation or function do not influence the behavioral output in this assay. More behavioral assays in the future might bring further insights into the involvement of different neuronal populations in phenotypic outputs.

Our main hypothesis regarding the regulated transcripts in the *lbx1a^(−/−)^* animals was that we should see differences, among others, in neuronally expressed transcripts, especially at the inhibitory/excitatory cell fate boundary. Unexpectedly, with the gene enrichment analysis, we were unable to detect any of these GO terms. Instead, among the downregulated genes, a potential chromosome organization phenotype and an altered RNA processing, splicing, or stability phenotype was found in *lbx1a^(−/−)^* animals. Lbx1a as a transcriptional regulator could therefore regulate RNA processing genes, which could influence neuronal differentiation via, e.g., alternative splicing, a phenomenon that is well known in neurons especially in GABAergic and glutamatergic differentiation [59]. On the other hand, gene enrichment analysis among the upregulated transcripts delivered in the molecular function category translation factor binding (GO:0008135) as well as several other related GO terms driven by the same gene set. Interestingly, increased translation could be associated with neurodevelopmental aspects of certain types of neurons [60]. Another reason why we did not detect more neuronal GO terms could be the type of sampling we applied. We cut the larvae as it was shown in Figure 4A. Therefore, only a portion of the tissue reflects brain tissues, the jaw region and maybe some internal organs were sampled as well, which might lead to a dilution of neuronal transcripts among other non-neuronal transcripts coming from other tissues. It could be that we missed important transcriptional regulation in neuronal cells due to this dilution effect because important transcripts were simply outnumbered by non-neuronal transcripts and therefore below detection threshold. The tail region, including the main part of the spinal cord, was used for genotyping so that neuronal transcripts from the spinal cord were not included in this assay.

To gain more insights into direct targets of LBX1 transcriptional activity, we overexpressed the human version of the gene in the HEK293 cell culture system and performed a similar RNAseq experiment. Although this cell line does not reflect all aspects of neuronal behavior in cell culture, we used this experiment to gain additional hints regarding which genes could potentially be regulated by LBX1. Due to the strong artificial overexpression, we identified 357 downregulated and 872 upregulated transcripts. Among the downregulated GO terms, we found the WNT signaling pathway (GO:0016055) and axonogenesis (GO:0007409) as biological processes affected due to overexpression. The WNT signaling pathway especially could be important to assess LBX1 function in neurodevelopment [61]. In addition, the pathway analysis delivered several neuronal items, e.g., neurodegeneration (hsa05022), Alzheimer’s (hsa05010), and oxytocin signaling pathway (hsa04921), indicating putative neuronal targets of LBX1. The combined analysis of cell culture overexpression and in vivo null mutant expression differences showed only two directional overlapping genes. Upregulated in mutant and downregulated in cell culture was *FBN3*, and downregulated in mutant and upregulated in cell culture was *INA*. *FBN3* (Fibrillin 3) is an extracellular matrix glycoprotein that assembles into microfibrils and plays important functions in connective tissue biology [62]. Mutations in *FBN3* as well as in two other family members of the fibrillin-family *FBN1* and *FBN2* have been identified in several disorders, including Bardet–Biedl syndrome and Marfan syndrome [63]. As this gene is upregulated in the mutant line and downregulated due to the overexpression of LBX1, we think that LBX1 maybe acts as a transcriptional repressor for this target gene. *INA* (alpha-Internexin) is a structural component of the neurofilament family and widely expressed in neurons and interconnect actin and microtubules, thus forming the neuronal cytoskeleton [64]. This gene was found to be downregulated in the mutant line and upregulated due to the overexpression of human LBX1, so we think that LBX1 maybe acts as a transcriptional activator for this target gene. How these two genes might be involved in the spectrum of LBX1-related human diseases and phenotypes requires further investigation.

The robust novelty-induced locomotor hyperactivity phenotype observed in both *lbx1a^(−/−)^* and lbx1b^(−/−)^ animals suggests that dysregulated LBX1 function is associated with a risk for ADHD, as indicated by the perfect segregation of a SNP variant (rs941909) in an extended pedigree with a high density of affected individuals [28]. While the synonymous SNP rs941909 is not changing the amino acid sequence and therefore the protein structure of LBX1, it may be in linkage disequilibrium with functionally active variant(s) in regulatory regions impacting the expression of the gene. Nevertheless, this notion of a link between LBX1 function and hyperactivity as a hallmark of ADHD is further supported by the evidence that Lbx1 is required for specification of GABAergic neuron cell fate, which involves also the expression of peptide neuromodulators, such as neuropeptide y (NPY) [4]. Neuropeptide Y has previously been implicated in ADHD [65] and emotional dysregulation, including anxiety [66]. Thus, alteration in GABAergic signaling may result in E/I imbalance, which has been reported for neurodevelopmental disorders and related traits [67]. Moreover, given that Lbx1 drives GABAergic interneuron development in the spinal cord and regulates muscle precursor cell migration, it is also plausible that Lbx1 dysfunction is not only involved in the pathogenesis of IS but also contributes to impaired motor coordination frequently (approx. 60%) observed in patients with ADHD.

In order to learn more about the mechanism how LBX1 could be linked to anxiety disorders, we extracted anxiety-related genes from several GWAS studies over the last few years [23,25,26,27] and compared the gene lists with the regulated genes we found in the mutant line and the overexpression experiment. Interestingly, we identified 13 overlapping genes regulated in the overexpression experiment and only three genes regulated in the *lbx1a^(−/−)^* mutant line (*NPM1*, *PCLO*, and *FADS2*), which are candidate genes from anxiety GWAS studies. *PCLO* especially is an interesting candidate as a member of the presynaptic active zone and therefore involved in vesicle release dynamics in neurons [68]. Besides anxiety disorders [69], *PCLO* plays a role in several other mental disorders like schizophrenia, post-traumatic stress disorder, depression, and many other disorders [70,71,72]. Therefore, *PCLO* could be an attractive gene to be investigated in our *lbx1a* and *lbx1b* mutant lines to further analyze its impact on anxiety-related phenotypes. However, further experimental validation of the expression changes is needed. In the future, we would like to validate LBX1-dependent gene regulation in human iPSCs by using CRISPR/Cas9 knock-out of *LBX1*, studying differentiation into neurons, and performing expression profiling.

## 5. Conclusions

The two newly developed zebrafish models of Lbx1 loss of function may be useful in future investigations to uncover the mechanistic background of behavioral phenotypes with relevance to psychiatric and somatic disorders related to human *LBX1* dysfunction. Double mutants, neuronal cell fate analysis, and characterization of adult animals could be tackled next. As several mental disorders, including various neurodevelopmental disorders, are thought to feature a disrupted equilibrium of excitation and inhibition in the nervous system of patients, known as E/I imbalance [73,74,75], a deeper understanding of this phenomenon will be gained in these newly generated mutant lines. In addition, an iPSC study on the differentiation of midbrain dopaminergic neurons has shown recently that LBX1 is involved in the promotion of differentiation of these neurons, probably via regulating cholesterol biosynthesis [76]. These aspects could be further addressed and validated in our mutant lines. Moreover, somatic disorders associated with LBX1 dysfunction could be explored further in these lines.

## Data Availability

All datasets generated for this study are included in the manuscript and the Supplementary Files. RNAseq raw data files are available upon reasonable request.

## Supplementary Materials

The following supporting information can be downloaded at https://www.mdpi.com/article/10.3390/cells14241980/s1, Figures S1–S10: Supplementary_Figures_1_10.pdf; Tables S1–S19: Supplementary_Tables_1_19.xlsx.

## References

[1] Jagla K., Dollé P., Mattei M.G., Jagla T., Schuhbaur B., Dretzen G., Bellard F., Bellard M. (1995). Mouse Lbx1 and human LBX1 define a novel mammalian homeobox gene family related to the Drosophila lady bird genes. Mech. Dev..

[2] Wotton K.R., Weierud F.K., Dietrich S., Lewis K.E. (2008). Comparative genomics of Lbx loci reveals conservation of identical Lbx ohnologs in bony vertebrates. BMC Evol. Biol..

[3] Gross M.K., Dottori M., Goulding M. (2002). Lbx1 Specifies Somatosensory Association Interneurons in the Dorsal Spinal Cord. Neuron.

[4] Huang M., Huang T., Xiang Y., Xie Z., Chen Y., Yan R., Xu J., Cheng L. (2008). Ptf1a, Lbx1 and Pax2 coordinate glycinergic and peptidergic transmitter phenotypes in dorsal spinal inhibitory neurons. Dev. Biol..

[5] Cheng L., Samad O.A., Xu Y., Mizuguchi R., Luo P., Shirasawa S., Goulding M., Ma Q. (2005). Lbx1 and Tlx3 are opposing switches in determining GABAergic versus glutamatergic transmitter phenotypes. Nat. Neurosci..

[6] Nadadhur A.G., Leferink P.S., Holmes D., Hinz L., Cornelissen-Steijger P., Gasparotto L., Heine V.M. (2018). Patterning factors during neural progenitor induction determine regional identity and differentiation potential in vitro. Stem Cell Res..

[7] Brohmann H., Jagla K., Birchmeier C. (2000). The role of Lbx1 in migration of muscle precursor cells. Development.

[8] Schäfer K., Braun T. (1999). Early specification of limb muscle precursor cells by the homeobox gene Lbx1h. Nat. Genet..

[9] Masselink W., Masaki M., Sieiro D., Marcelle C., Currie P.D. (2017). Phosphorylation of Lbx1 controls lateral myoblast migration into the limb. Dev. Biol..

[10] Rinsky L.A., Gamble J.G. (1988). Adolescent idiopathic scoliosis. West. J. Med..

[11] De Salvatore S., Ruzzini L., Longo U.G., Marino M., Greco A., Piergentili I., Costici P.F., Denaro V. (2022). Exploring the association between specific genes and the onset of idiopathic scoliosis: A systematic review. BMC Med. Genom..

[12] Fan Y.H., Song Y.Q., Chan D., Takahashi Y., Ikegawa S., Matsumoto M., Kou I., Cheah K.S., Sham P., Cheung K.M. (2012). SNP rs11190870 near LBX1 is associated with adolescent idiopathic scoliosis in southern Chinese. J. Hum. Genet..

[13] Jiang H., Qiu X., Dai J., Yan H., Zhu Z., Qian B., Qiu Y. (2013). Association of rs11190870 near LBX1 with adolescent idiopathic scoliosis susceptibility in a Han Chinese population. Eur. Spine J..

[14] Zhu Z., Tang N.L., Xu L., Qin X., Mao S., Song Y., Liu L., Li F., Liu P., Yi L. (2015). Genome-wide association study identifies new susceptibility loci for adolescent idiopathic scoliosis in Chinese girls. Nat. Commun..

[15] Luo M., Zhang Y., Huang S., Song Y. (2020). The Susceptibility and Potential Functions of the LBX1 Gene in Adolescent Idiopathic Scoliosis. Front. Genet..

[16] Al Mekkawi A.K., Caruso J.P., El Ahmadieh T.Y., Palmisciano P., Aljardali M.W., Derian A.G., Al Tamimi M., Bagley C.A., Aoun S.G. (2023). Single Nucleotide Polymorphisms and Adolescent Idiopathic Scoliosis: A Systematic Review and Meta-analysis of the Literature. Spine (Phila Pa 1976).

[17] Guo L., Yamashita H., Kou I., Takimoto A., Meguro-Horike M., Horike S., Sakuma T., Miura S., Adachi T., Yamamoto T. (2016). Functional Investigation of a Non-coding Variant Associated with Adolescent Idiopathic Scoliosis in Zebrafish: Elevated Expression of the Ladybird Homeobox Gene Causes Body Axis Deformation. PLoS Genet..

[18] Janusz P., Tokłowicz M., Andrusiewicz M., Kotwicka M., Kotwicki T. (2022). Association of LBX1 Gene Methylation Level with Disease Severity in Patients with Idiopathic Scoliosis: Study on Deep Paravertebral Muscles. Genes.

[19] Holder-Espinasse M., Jamsheer A., Escande F., Andrieux J., Petit F., Sowinska-Seidler A., Socha M., Jakubiuk-Tomaszuk A., Gerard M., Mathieu-Dramard M. (2019). Duplication of 10q24 locus: Broadening the clinical and radiological spectrum. Eur. J. Hum. Genet..

[20] Cova G., Glaser J., Schöpflin R., Prada-Medina C.A., Ali S., Franke M., Falcone R., Federer M., Ponzi E., Ficarella R. (2023). Combinatorial effects on gene expression at the Lbx1/Fgf8 locus resolve split-hand/foot malformation type 3. Nat. Commun..

[21] Hernandez-Miranda L.R., Ibrahim D.M., Ruffault P.L., Larrosa M., Balueva K., Muller T., Weerd W., Stolte-Dijkstra I., Hostra R.M.W., Brunet J.F. (2018). Mutation in LBX1/Lbx1 precludes transcription factor cooperativity and causes congenital hypoventilation in humans and mice. Proc. Natl. Acad. Sci. USA.

[22] Pagliardini S., Ren J., Gray P.A., Vandunk C., Gross M., Goulding M., Greer J.J. (2008). Central respiratory rhythmogenesis is abnormal in lbx1- deficient mice. J. Neurosci..

[23] Otowa T., Hek K., Lee M., Byrne E.M., Mirza S.S., Nivard M.G., Bigdeli T., Aggen S.H., Adkins D., Wolen A. (2016). Meta-analysis of genome-wide association studies of anxiety disorders. Mol. Psychiatry.

[24] Hettema J.M., Verhulst B., Chatzinakos C., Bacanu S.A., Chen C.Y., Ursano R.J., Kessler R.C., Gelernter J., Smoller J.W., He F. (2020). Genome-wide association study of shared liability to anxiety disorders in Army STARRS. Am. J. Med. Genet. B Neuropsychiatr. Genet..

[25] Friligkou E., Løkhammer S., Cabrera-Mendoza B., Shen J., He J., Deiana G., Zanoaga M.D., Asgel Z., Pilcher A., Di Lascio L. (2024). Gene discovery and biological insights into anxiety disorders from a large-scale multi-ancestry genome-wide association study. Nat. Genet..

[26] Strom N.I., Verhulst B., Bacanu S., Cheesman R., Purves K.L., Gedik H., Mitchell B.L., Kwong A.S., Faucon A.B., Singh K. (2024). Genome-wide association study of major anxiety disorders in 122,341 European-ancestry cases identifies 58 loci and highlights GABAergic signaling. medRxiv.

[27] Tesfaye M., Jaholkowski P., Shadrin A.A., van der Meer D., Hindley G.F.L., Holen B., Parker N., Parekh P., Birkenæs V., Rahman Z. (2024). Identification of novel genomic loci for anxiety symptoms and extensive genetic overlap with psychiatric disorders. Psychiatry Clin. Neurosci..

[28] Schäfer N., Friedrich M., Jørgensen M., Kollert S., Koepsell H., Wischmeyer E., Lesch K.-P., Geiger D., Döring F. (2018). Functional analysis of a triplet deletion in the gene encoding the sodium glucose transporter 3, a potential risk factor for ADHD. PLoS ONE.

[29] Godinho L. (2011). Imaging Zebrafish Development. Cold Spring Harb. Protoc..

[30] Kimmel C.B., Ballard W.W., Kimmel S.R., Ullmann B., Schilling T.F. (1995). Stages of embryonic development of the zebrafish. Dev. Dyn..

[31] Labun K., Montague T.G., Krause M., Torres Cleuren Y.N., Tjeldnes H., Valen E. (2019). CHOPCHOP v3: Expanding the CRISPR web toolbox beyond genome editing. Nucleic Acids Res..

[32] Love M.I., Huber W., Anders S. (2014). Moderated estimation of fold change and dispersion for RNA-seq data with DESeq2. Genome Biol..

[33] Yu G., Wang L.G., Han Y., He Q.Y. (2012). clusterProfiler: An R package for comparing biological themes among gene clusters. Omics.

[34] Luo W., Brouwer C. (2013). Pathview: An R/Bioconductor package for pathway-based data integration and visualization. Bioinformatics.

[35] Tang D., Chen M., Huang X., Zhang G., Zeng L., Zhang G., Wu S., Wang Y. (2023). SRplot: A free online platform for data visualization and graphing. PLoS ONE.

[36] JASP (2025). JASP (Version 0.95.4). https://jasp-stats.org/.

[37] Faul F., Erdfelder E., Lang A.G., Buchner A. (2007). G*Power 3: A flexible statistical power analysis program for the social, behavioral, and biomedical sciences. Behav. Res. Methods.

[38] Faul F., Erdfelder E., Buchner A., Lang A.G. (2009). Statistical power analyses using G*Power 3.1: Tests for correlation and regression analyses. Behav. Res. Methods.

[39] Lukowski C.M., Drummond D.L., Waskiewicz A.J. (2011). Pbx-dependent regulation of lbx gene expression in developing zebrafish embryos. Genome.

[40] Juárez-Morales J.L., Weierud F., England S.J., Demby C., Santos N., Grieb G., Mazan S., Lewis K.E. (2021). Evolution of lbx spinal cord expression and function. Evol. Dev..

[41] Richendrfer H., Pelkowski S.D., Colwill R.M., Creton R. (2012). On the edge: Pharmacological evidence for anxiety-related behavior in zebrafish larvae. Behav. Brain Res..

[42] Julien A., Perrin S., Duchamp de Lageneste O., Carvalho C., Bensidhoum M., Legeai-Mallet L., Colnot C. (2020). FGFR3 in Periosteal Cells Drives Cartilage-to-Bone Transformation in Bone Repair. Stem Cell Rep..

[43] Sun X., Zhang R., Chen H., Du X., Chen S., Huang J., Liu M., Xu M., Luo F., Jin M. (2020). Fgfr3 mutation disrupts chondrogenesis and bone ossification in zebrafish model mimicking CATSHL syndrome partially via enhanced Wnt/β-catenin signaling. Theranostics.

[44] Putnam E.A., Zhang H., Ramirez F., Milewicz D.M. (1995). Fibrillin-2 (FBN2) mutations result in the Marfan-like disorder, congenital contractural arachnodactyly. Nat. Genet..

[45] Buchan J.G., Alvarado D.M., Haller G.E., Cruchaga C., Harms M.B., Zhang T., Willing M.C., Grange D.K., Braverman A.C., Miller N.H. (2014). Rare variants in FBN1 and FBN2 are associated with severe adolescent idiopathic scoliosis. Hum. Mol. Genet..

[46] Sheng F., Xia C., Xu L., Qin X., Tang N.L., Qiu Y., Cheng J.C., Zhu Z. (2019). New Evidence Supporting the Role of FBN1 in the Development of Adolescent Idiopathic Scoliosis. Spine (Phila Pa 1976).

[47] Powers R.M., Daza R., Koehler A.E., Courchet J., Calabrese B., Hevner R.F., Halpain S. (2022). Growth cone macropinocytosis of neurotrophin receptor and neuritogenesis are regulated by neuron navigator 1. Mol. Biol. Cell.

[48] Paronett E.M., Meechan D.W., Karpinski B.A., LaMantia A.S., Maynard T.M. (2015). Ranbp1, Deleted in DiGeorge/22q11.2 Deletion Syndrome, is a Microcephaly Gene That Selectively Disrupts Layer 2/3 Cortical Projection Neuron Generation. Cereb. Cortex.

[49] Barriga E.H., Alasaadi D.N., Mencarelli C., Mayor R., Pichaud F. (2022). RanBP1 plays an essential role in directed migration of neural crest cells during development. Dev. Biol..

[50] Xie Y., Lv X., Ni D., Liu J., Hu Y., Liu Y., Liu Y., Liu R., Zhao H., Lu Z. (2019). HPD degradation regulated by the TTC36-STK33-PELI1 signaling axis induces tyrosinemia and neurological damage. Nat. Commun..

[51] Heinonen J., Taipaleenmäki H., Roering P., Takatalo M., Harkness L., Sandholm J., Uusitalo-Järvinen H., Kassem M., Kiviranta I., Laitala-Leinonen T. (2011). Snorc is a novel cartilage specific small membrane proteoglycan expressed in differentiating and articular chondrocytes. Osteoarthr. Cartil..

[52] Heinonen J., Zhang F.P., Surmann-Schmitt C., Honkala S., Stock M., Poutanen M., Säämänen A.M. (2017). Defects in chondrocyte maturation and secondary ossification in mouse knee joint epiphyses due to Snorc deficiency. Osteoarthr. Cartil..

[53] Kolberg L., Raudvere U., Kuzmin I., Adler P., Vilo J., Peterson H. (2023). g:Profiler—Interoperable web service for functional enrichment analysis and gene identifier mapping (2023 update). Nucleic Acids Res..

[54] Bardou P., Mariette J., Escudié F., Djemiel C., Klopp C. (2014). jvenn: An interactive Venn diagram viewer. BMC Bioinform..

[55] van der Laan C.M., Ip H.F., Schipper M., Hottenga J.-J., St Pourcain B., Zayats T., Pool R., Krapohl E.M.L., Brikell I., Soler Artigas M. (2025). Genome-wide association meta-analysis of childhood ADHD symptoms and diagnosis identifies new loci and potential effector genes. Nat. Genet..

[56] Ochi H., Westerfield M. (2009). Lbx2 regulates formation of myofibrils. BMC Dev. Biol..

[57] Lou Q., He J., Hu L., Yin Z. (2012). Role of lbx2 in the noncanonical Wnt signaling pathway for convergence and extension movements and hypaxial myogenesis in zebrafish. Biochim. Biophys. Acta.

[58] Talbot J.C., Teets E.M., Ratnayake D., Duy P.Q., Currie P.D., Amacher S.L. (2019). Muscle precursor cell movements in zebrafish are dynamic and require Six family genes. Development.

[59] Feng H., Moakley D.F., Chen S., McKenzie M.G., Menon V., Zhang C. (2021). Complexity and graded regulation of neuronal cell-type–specific alternative splicing revealed by single-cell RNA sequencing. Proc. Natl. Acad. Sci. USA.

[60] Wefers Z., Alecki C., Huang R., Jacob-Tomas S., Vera M. (2022). Analysis of the Expression and Subcellular Distribution of eEF1A1 and eEF1A2 mRNAs during Neurodevelopment. Cells.

[61] Rosso S.B., Inestrosa N.C., Rosso S.B. (2013). WNT signaling in neuronal maturation and synaptogenesis. Front. Cell. Neurosci..

[62] Summers K.M. (2024). Genetic models of fibrillinopathies. Genetics.

[63] Genovesi M.L., Torres B., Goldoni M., Salvo E., Cesario C., Majolo M., Mazza T., Piscopo C., Bernardini L. (2022). Case Report: A Novel Homozygous Missense Variant of FBN3 Supporting It Is a New Candidate Gene Causative of a Bardet-Biedl Syndrome-Like Phenotype. Front. Genet..

[64] Kotaich F., Caillol D., Bomont P. (2023). Neurofilaments in health and Charcot-Marie-Tooth disease. Front. Cell Dev. Biol..

[65] Lesch K.P., Selch S., Renner T.J., Jacob C., Nguyen T.T., Hahn T., Romanos M., Walitza S., Shoichet S., Dempfle A. (2011). Genome-wide copy number variation analysis in attention-deficit/hyperactivity disorder: Association with neuropeptide Y gene dosage in an extended pedigree. Mol. Psychiatry.

[66] Bale R., Doshi G. (2023). Cross talk about the role of Neuropeptide Y in CNS disorders and diseases. Neuropeptides.

[67] Lesch K.P., Gorbunov N. (2025). Antisocial personality disorder:Failure to balance excitation/inhibition?. Neuropharmacology.

[68] Ivanova D., Dirks A., Fejtova A. (2016). Bassoon and piccolo regulate ubiquitination and link presynaptic molecular dynamics with activity-regulated gene expression. J. Physiol..

[69] Li W., Chen R., Feng L., Dang X., Liu J., Chen T., Yang J., Su X., Lv L., Li T. (2024). Genome-wide meta-analysis, functional genomics and integrative analyses implicate new risk genes and therapeutic targets for anxiety disorders. Nat. Hum. Behav..

[70] Chick S.L., Holmans P., Cameron D., Grozeva D., Sims R., Williams J., Bray N.J., Owen M.J., O’Donovan M.C., Walters J.T.R. (2025). Whole-exome sequencing analysis identifies risk genes for schizophrenia. Nat. Commun..

[71] Nievergelt C.M., Maihofer A.X., Atkinson E.G., Chen C.Y., Choi K.W., Coleman J.R.I., Daskalakis N.P., Duncan L.E., Polimanti R., Aaronson C. (2024). Genome-wide association analyses identify 95 risk loci and provide insights into the neurobiology of post-traumatic stress disorder. Nat. Genet..

[72] Mbarek H., Milaneschi Y., Hottenga J.J., Ligthart L., de Geus E.J.C., Ehli E.A., Willemsen G., Davies G.E., Smit J.H., Boomsma D.I. (2017). Genome-Wide Significance for PCLO as a Gene for Major Depressive Disorder. Twin Res. Hum. Genet..

[73] Canitano R., Pallagrosi M. (2017). Autism Spectrum Disorders and Schizophrenia Spectrum Disorders: Excitation/Inhibition Imbalance and Developmental Trajectories. Front. Psychiatry.

[74] Lopatina O.L., Malinovskaya N.A., Komleva Y.K., Gorina Y.V., Shuvaev A.N., Olovyannikova R.Y., Belozor O.S., Belova O.A., Higashida H., Salmina A.B. (2019). Excitation/inhibition imbalance and impaired neurogenesis in neurodevelopmental and neurodegenerative disorders. Rev. Neurosci..

[75] Mamiya P.C., Arnett A.B., Stein M.A. (2021). Precision Medicine Care in ADHD: The Case for Neural Excitation and Inhibition. Brain Sci..

[76] Gomez Ramos B., Ohnmacht J., de Lange N., Valceschini E., Ginolhac A., Catillon M., Ferrante D., Rakovic A., Halder R., Massart F. (2024). Multiomics analysis identifies novel facilitators of human dopaminergic neuron differentiation. EMBO Rep..

