# Supplementary material for: Hyperactivity and Differential Gene Expression in lbx1a(−/−) Zebrafish Larvae
Source: Cells. 2025 Dec 12;14(24):1980. doi: 10.3390/cells14241980 (PMC12731876; doi:10.3390/cells14241980)
Supplement: Supplementary file 1 [file cells-14-01980-s001.zip › Supplementary_Figures_editable.pdf]

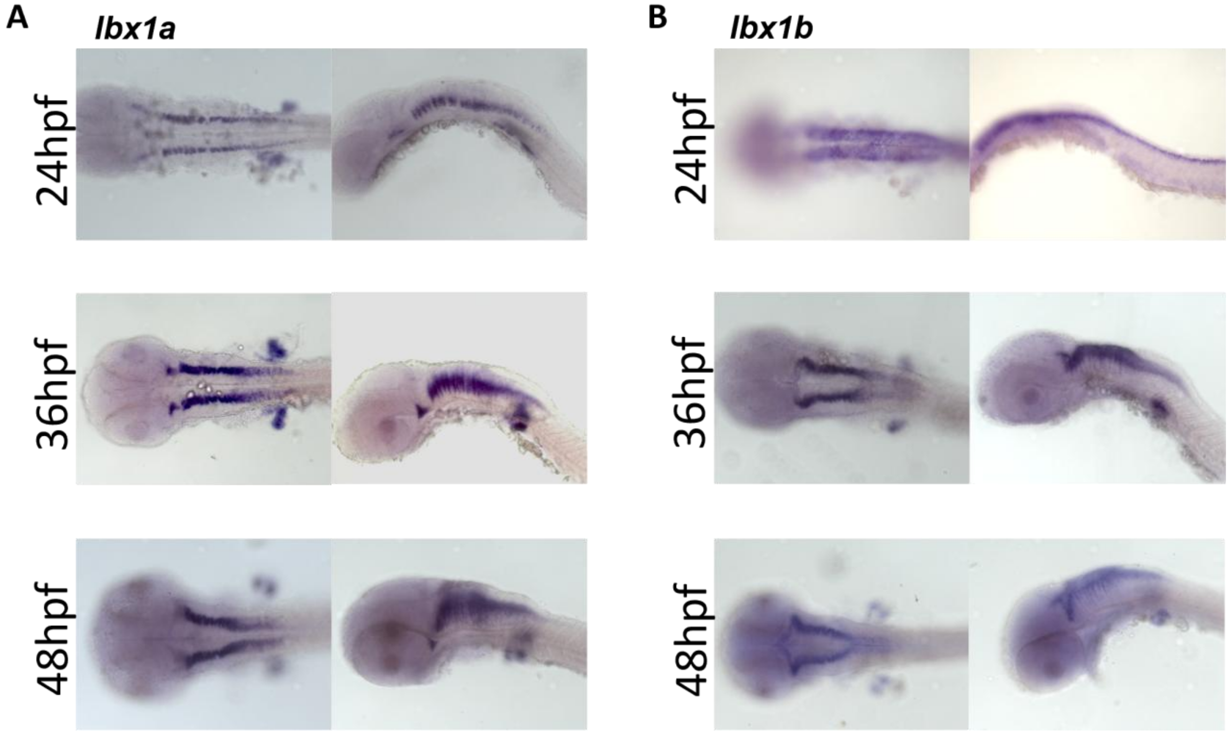

Supplementary Figure S1. Both zebrafish orthologs are expressed in overlapping as well as distinct expression domains. (**A**) Expression pattern of *lbx1a* transcripts in wildtype zebrafish larvae at 24hpf, 36hpf, and 48hpf (from top to bottom) are shown with in situ hybridizations. Dorsal (left) and lateral views (right) are displayed. **(B)** Expression pattern of *lbx1b* transcripts in wildtype zebrafish larvae at 24hpf, 36hpf, and 48hpf (from top to bottom) are shown with in situ hybridizations. Dorsal (left) and lateral views (right) are displayed.

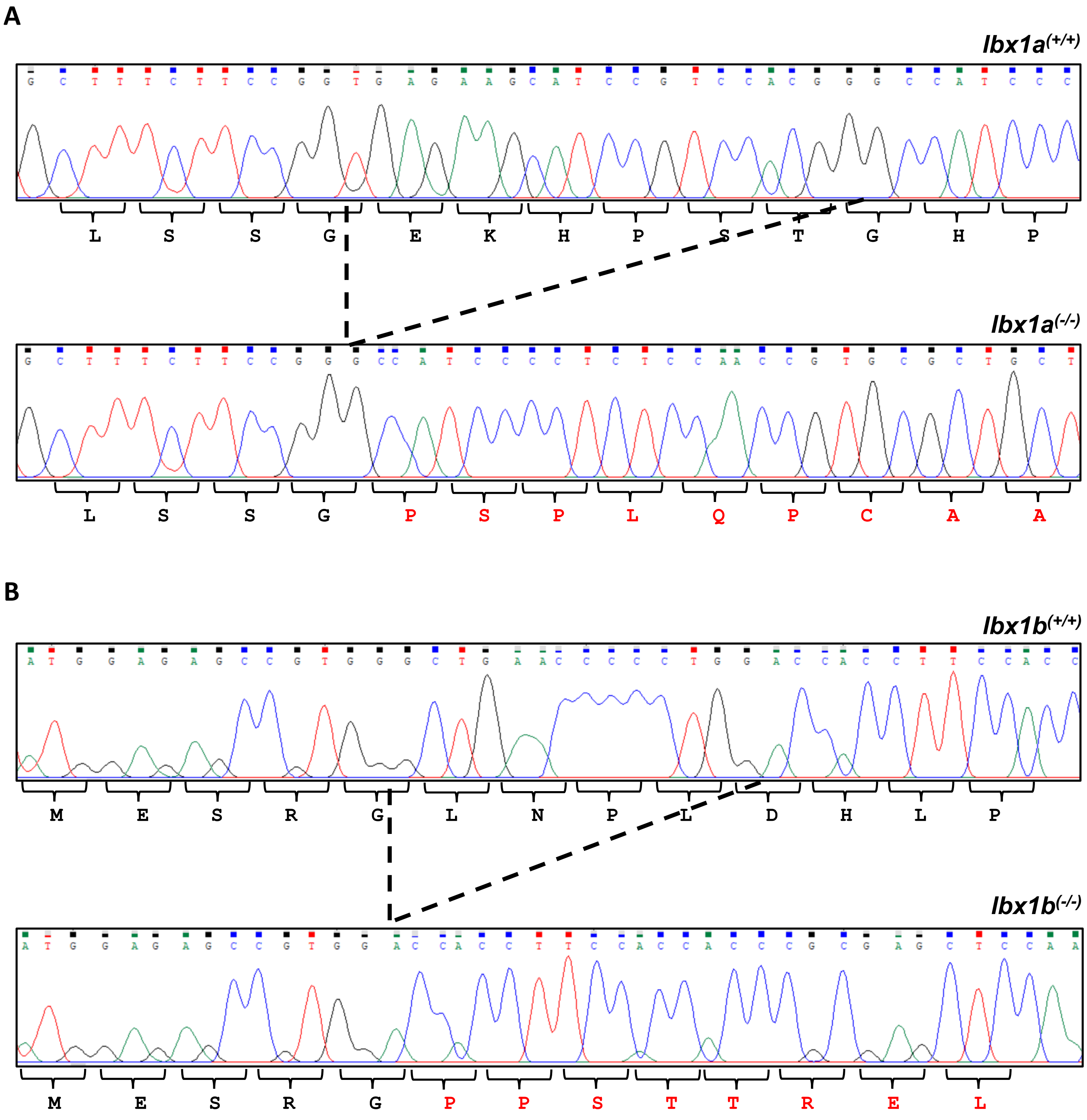

Supplementary Figure S2. Genetic details of *lbx1a* and *lbx1b* mutant lines. (**A**) DNA sequencing traces for genotyping PCR products of *lbx1a*$^{(+/+)}$ and *lbx1a*$^{(-/-)}$ mutant animals. *lbx1a*$^{(-/-)}$ traces show a loss of 20 nt in total. Translated amino acid sequence is annotated below, and frameshifted sequence is marked in red. **(B)** DNA sequencing traces for genotyping PCR product of *lbx1b*$^{(+/+)}$ and *lbx1b*$^{(-/-)}$ mutant animals. *lbx1b*$^{(-/-)}$ traces show a loss of 14 nt in total. Translated amino acid sequence is annotated below, and frameshifted sequence is marked in red.

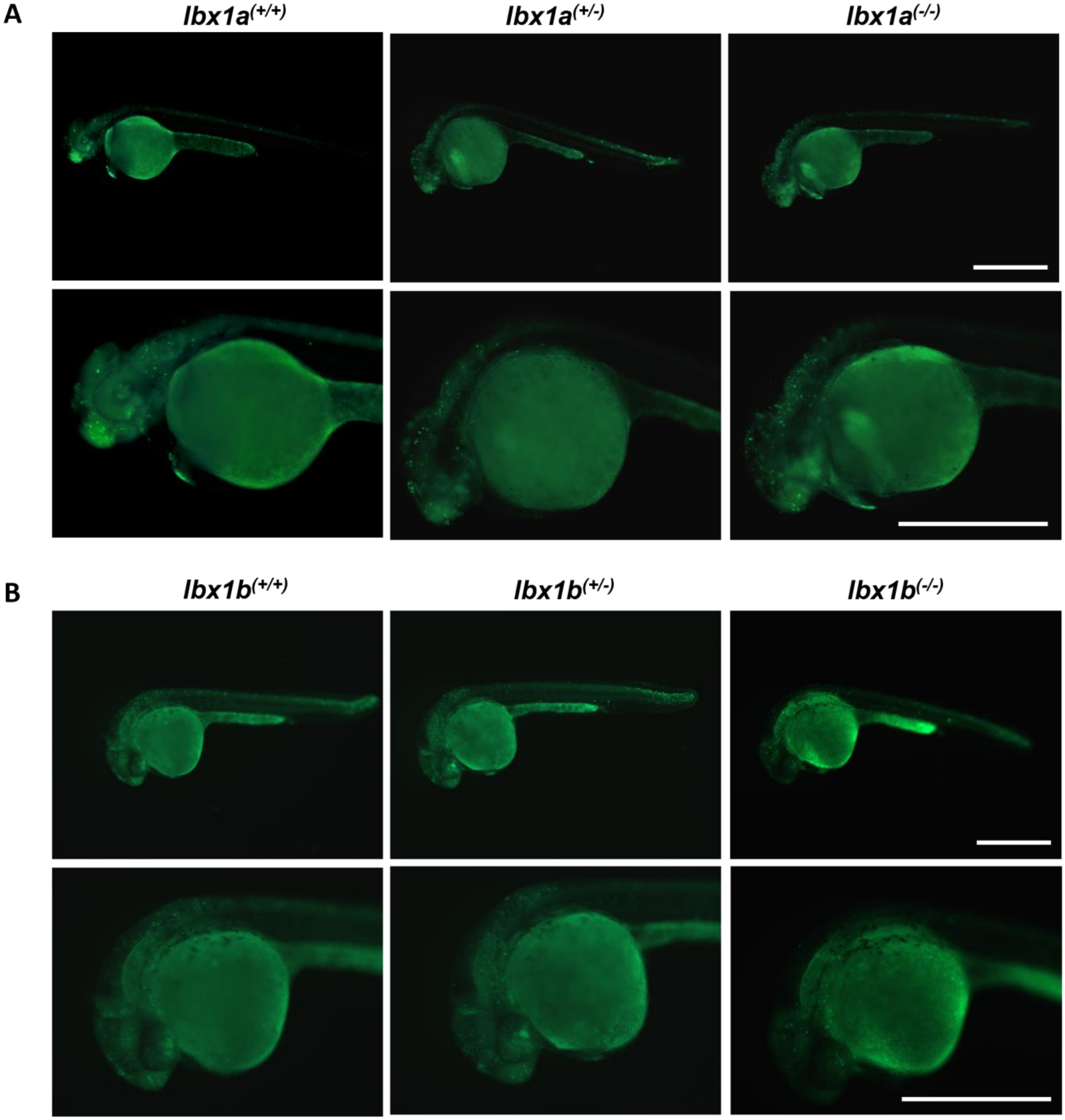

Supplementary Figure S3. No evidence for increased cell death in *lbx1a* and *lbx1b* mutants. (**A**) Acridine orange stainings in *lbx1a*$^{(+/+)}$, *lbx1a*$^{(+/-)}$ and *lbx1a*$^{(-/-)}$ mutant larvae (from left to right). Enlarged pictures of the first row are shown in the second row. **(B)** Acridine orange stainings in *lbx1b*$^{(+/+)}$, *lbx1b*$^{(+/-)}$ and *lbx1b*$^{(-/-)}$ mutant larvae (from left to right). Enlarged pictures of the first row are shown in the second row. Scale bar corresponds to 200µm.

A

lbx1a	+/+	+/-	-/-
Observed	108	209	103
Expected	105	210	105

B

lbx1b	+/+	+/-	-/-
Observed	102	198	128
Expected	107	214	107

Supplementary Figure S4. Analysis of genotype ratios in *lbx1a* and *lbx1b* lines at 5 dpf. (**A**) *lbx1a* mutant line show expected mendelian ratios. **(B)** *lbx1b* mutant line show expected mendelian ratios. Genotype data were accumulated over three clutches in each line.

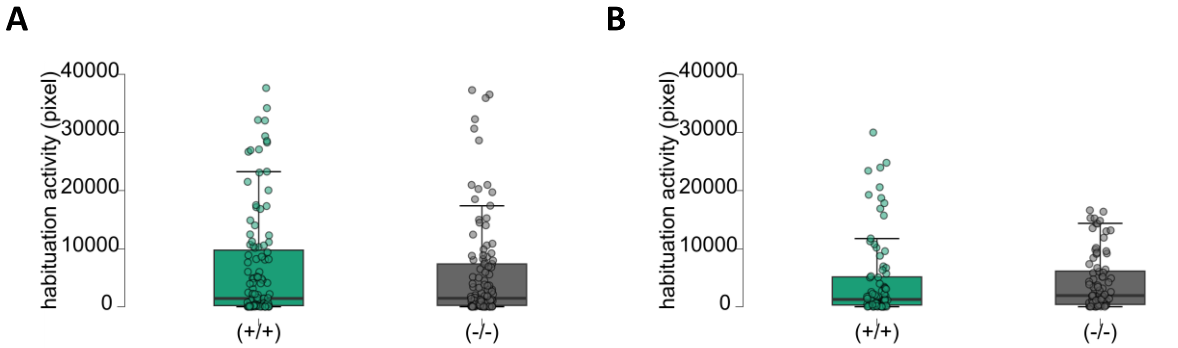

Supplementary Figure S5. No differences in habituation activity in *lbx1a* and *lbx1b* mutant lines. (**A**) Analysis of habituation activity in the *lbx1a* mutant line showed no difference compared to wildtype littermates (Mann-Whitney, p = 0.325). **(B)** Analysis of habituation activity in the *lbx1b* mutant line showed no difference compared to wildtype littermates (Mann-Whitney, p = 0.790).

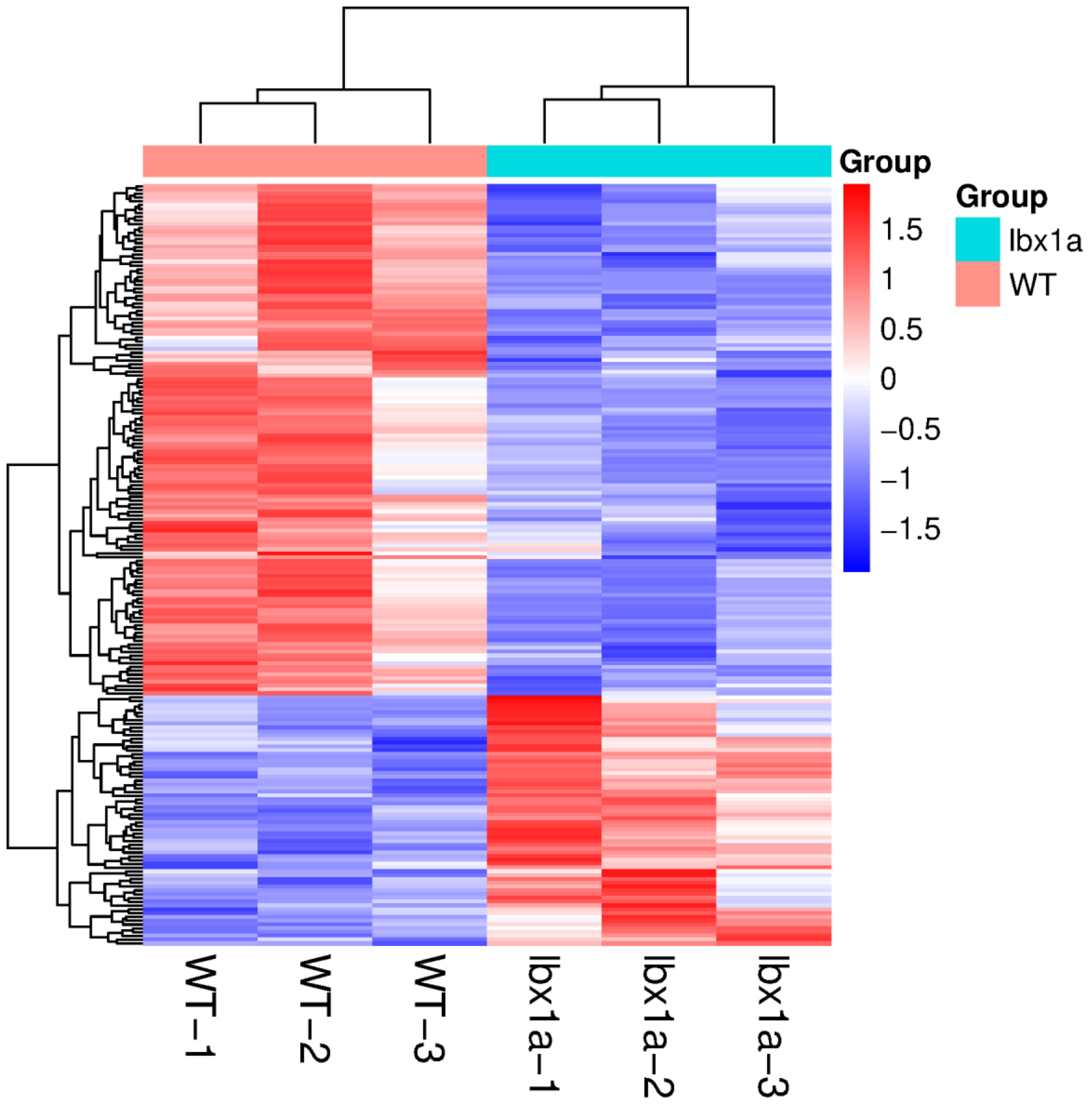

Supplementary Figure S6. Heatmap of differential expressed transcripts in *lbx1a*$^{(-/-)}$ mutant larvae. Mutant and wildtype samples cluster together, respectively. One hundred thirty-five DEGs were upregulated and sixty-six downregulated. Note the clustering of genotypes thus confirming reliability between the samples.

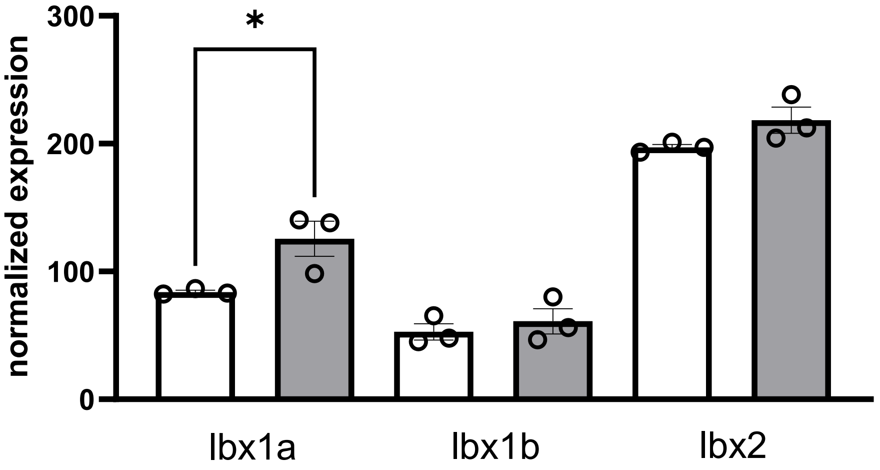

Supplementary Figure S7. Expression of *lbx*-family members in *lbx1a*$^{(-/-)}$ mutant larvae as measured with RNAseq. Note the slightly elevated expression of *lbx1a* in mutant samples. Only nominal significance was achieved thus confirming the qPCR results.

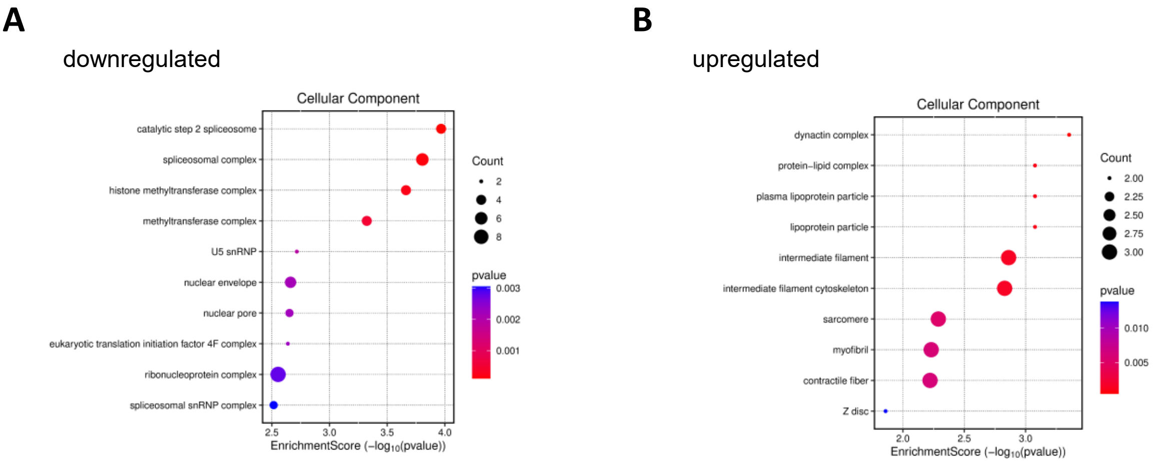

Supplementary Figure S8. GO term analysis in *lbx1a*$^{(-/-)}$ animals. **(A)** GO term enrichment of downregulated genes, cellular component. **(B)** GO term enrichment of upregulated genes: cellular component.

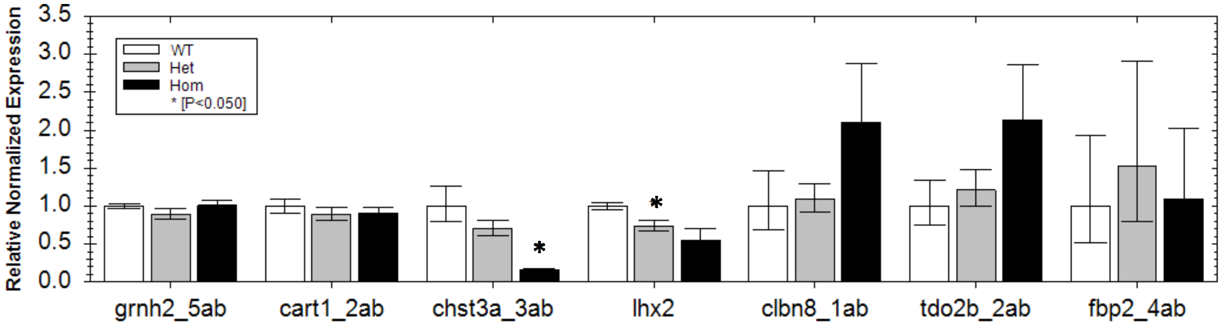

Supplementary Figure S9. Validation of expression differences in *lbx1*$^{(-/-)}$ mutant larvae with qPCR. Expression was normalized to *gapdh* and *b-actin* as housekeeping genes. *gnrh2* and *cart1* didn't show regulation, whereas *chst3a* and *lhx2* are downregulated in *lbx1*$^{(-/-)}$ mutant larvae. *Tdo2b* and *cbln8* are upregulated. * p > 0.05 unpaired t-test.

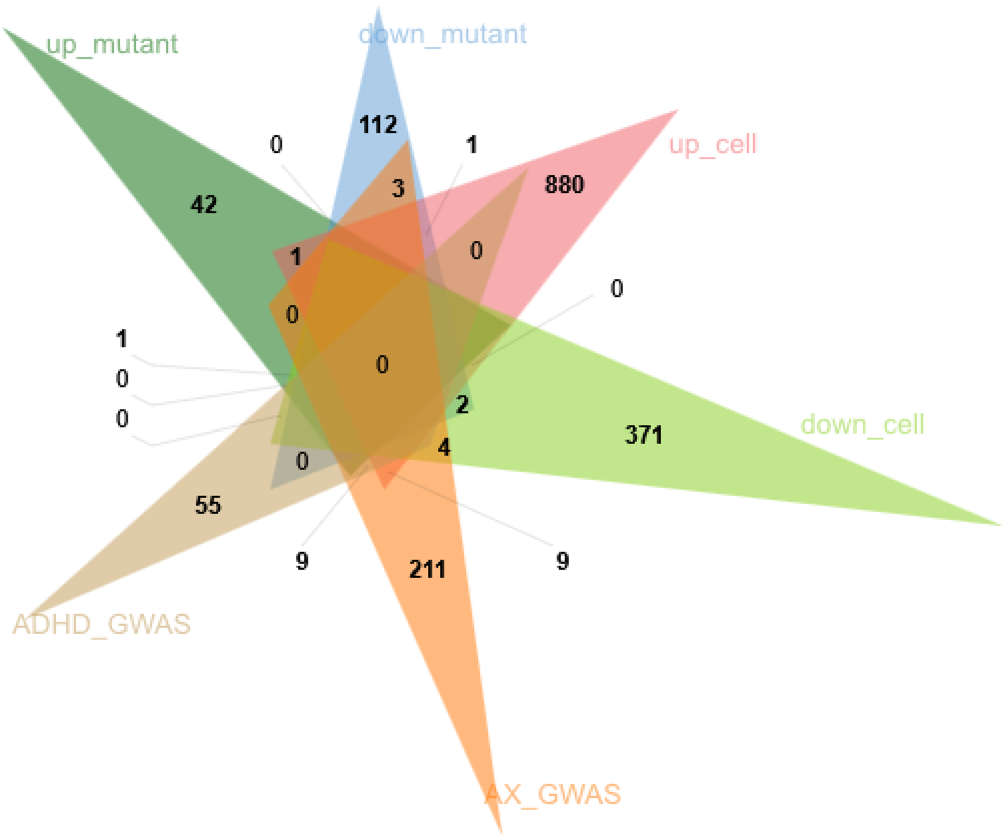

Supplementary Figure S10. Combined analysis of DEGs in both experimental set-ups and GWAS candidate genes for anxiety disorder and ADHD. Venn diagram showing the amount of overlap of the downregulated genes in *lbx1a*$^{(-/-)}$ animals (down_mutant, blue), upregulated in *lbx1a*$^{(-/-)}$ animals (up_mutant, dark_green), downregulated in HEK293 with LBX1 overexpression (down_cell, light green), upregulated in LBX1 overexpression (up_cell, red), GWAS candidate genes for anxiety disorders (AX_GWAS, orange) and GWAS candidate genes for ADHD (ADHD_GWAS, beige). Interestingly, there was an overlap of 9 genes common in both ADHD_GWAS and AX_GWAS (*MAML3, FBXL17, SORCS3, DCC, IP6K1, CDHR4, SEMA3F, UBA7,* and *FOXP2*).
